# Identification of *STXBP2* as a novel susceptibility locus for myocardial infarction in Japanese individuals by an exome-wide association study

**DOI:** 10.18632/oncotarget.16536

**Published:** 2017-03-23

**Authors:** Yoshiji Yamada, Jun Sakuma, Ichiro Takeuchi, Yoshiki Yasukochi, Kimihiko Kato, Mitsutoshi Oguri, Tetsuo Fujimaki, Hideki Horibe, Masaaki Muramatsu, Motoji Sawabe, Yoshinori Fujiwara, Yu Taniguchi, Shuichi Obuchi, Hisashi Kawai, Shoji Shinkai, Seijiro Mori, Tomio Arai, Masashi Tanaka

**Affiliations:** ^1^ Department of Human Functional Genomics, Advanced Science Research Promotion Center, Mie University, Tsu, Japan; ^2^ CREST, Japan Science and Technology Agency, Kawaguchi, Japan; ^3^ Computer Science Department, College of Information Science, University of Tsukuba, Tsukuba, Japan; ^4^ RIKEN Center for Advanced Intelligence Project, Tokyo, Japan; ^5^ Department of Computer Science, Nagoya Institute of Technology, Nagoya, Japan; ^6^ Department of Internal Medicine, Meitoh Hospital, Nagoya, Japan; ^7^ Department of Cardiology, Kasugai Municipal Hospital, Kasugai, Japan; ^8^ Department of Cardiovascular Medicine, Inabe General Hospital, Inabe, Japan; ^9^ Department of Cardiovascular Medicine, Gifu Prefectural Tajimi Hospital, Tajimi, Japan; ^10^ Department of Molecular Epidemiology, Medical Research Institute, Tokyo Medical and Dental University, Tokyo, Japan; ^11^ Section of Molecular Pathology, Graduate School of Health Care Sciences, Tokyo Medical and Dental University, Tokyo, Japan; ^12^ Research Team for Social Participation and Community Health, Tokyo Metropolitan Institute of Gerontology, Tokyo, Japan; ^13^ Research Team for Promoting Support System for Home Care, Tokyo Metropolitan Institute of Gerontology, Tokyo, Japan; ^14^ Research Team for Social Participation and Health Promotion, Tokyo Metropolitan Institute of Gerontology, Tokyo, Japan; ^15^ Center for Promotion of Clinical Investigation, Tokyo Metropolitan Geriatric Hospital, Tokyo, Japan; ^16^ Department of Pathology, Tokyo Metropolitan Geriatric Hospital, Tokyo, Japan; ^17^ Department of Clinical Laboratory, Tokyo Metropolitan Geriatric Hospital, Tokyo, Japan

**Keywords:** myocardial infarction, coronary artery disease, genetics, polymorphism, exome-wide association study

## Abstract

We performed exome-wide association studies to identify genetic variants—in particular, low-frequency variants with a large effect size—that confer susceptibility to coronary artery disease or myocardial infarction in Japanese. The exome-wide association studies were performed with 12,698 individuals (3488 subjects with coronary artery disease including 2438 with myocardial infarction, 9210 controls) and with the use of the Illumina HumanExome-12 DNA Analysis or Infinium Exome-24 BeadChip. The relation of allele frequencies for 41,339 single nucleotide polymorphisms that passed quality control to coronary artery disease or myocardial infarction was examined with Fisher's exact test. The exome-wide association study for coronary artery disease revealed that 126 single nucleotide polymorphisms were significantly (*P* <1.21 × 10^−6^) associated with this condition. Multivariable logistic regression analysis with adjustment for age, sex, and the prevalence of hypertension, diabetes mellitus, and dyslipidemia showed that six of these polymorphisms were related (*P* < 0.01) to coronary artery disease, but none was significantly (*P* < 9.92 × 10^−5^) associated with this condition. The exome-wide association study for myocardial infarction revealed that 114 single nucleotide polymorphisms were significantly (*P* <1.21 × 10^−6^) associated with this condition. Multivariable logistic regression analysis with adjustment for covariates revealed that nine of these polymorphisms were related (*P* < 0.01) to myocardial infarction. Among these nine polymorphisms, rs188212047 [G/T (L212F)] of *STXBP2* was significantly (dominant model; *P* = 4.84 × 10^−8^; odds ratio, 2.94) associated with myocardial infarction. *STXBP2* may thus be a novel susceptibility locus for myocardial infarction in Japanese.

## INTRODUCTION

Coronary artery disease (CAD) is a serious clinical problem because of its large contribution to mortality. Disease prevention is an important strategy for reducing the overall burden of CAD, and the identification of biomarkers for disease risk is key both for risk prediction and for potential intervention to reduce the chance of future coronary events.

Recent studies have highlighted the importance of genetic factors and of interactions between multiple genes and environmental factors in CAD [1]. The heritability of CAD was estimated to be 40% to 50% on the basis of family and twin studies [2]. Recent genome-wide association studies (GWASs) in European ancestry populations [3–8], African Americans [9], or Han Chinese [10, 11] have identified various genes and loci that confer susceptibility to CAD or myocardial infarction (MI). A meta-analysis of GWASs for CAD in European ancestry populations identified 46 loci with a genome-wide significance level and 104 variants at a false discovery rate of <5% [12]. These genetic variants typically have a minor allele frequency (MAF) of ≥5% and a small individual effect size, and they collectively account for only ~10.6% of the heritability of CAD [12]. A more recent meta-analysis for CAD among European ancestry populations that included low-frequency (0.5% ≤ MAF < 5%) variants identified 58 loci with a genome-wide significance level and 202 independent variants at a false discovery rate of <5% [13]. These genetic variants together account for ~28% of the heritability of CAD, showing that genetic susceptibility to this condition is largely determined by common variants with small effect sizes [13, 14]. Although several polymorphisms have been found to be significantly associated with MI in Japanese individuals [15, 16], genetic variants including low-frequency and rare variants, that contribute to genetic susceptibility to CAD or MI in Japanese remain to be identified definitively.

We have now performed exome-wide association studies (EWASs) with the use of exome array–based genotyping methods in order to identify genetic variants—in particular, low-frequency or rare coding variants with moderate to large effect sizes—that confer susceptibility to CAD or MI in Japanese. Given that most low-frequency or rare variants were not included in GWAS arrays of previous studies, we used Illumina arrays that provide coverage of putative functional single nucleotide polymorphisms (SNPs) in entire exons including low-frequency and rare variants.

## RESULTS

### Characteristics of subjects

The characteristics of the 12,698 subjects enrolled in the study are shown in Table 1. Age, the frequency of men, body mass index, and the prevalence of hypertension, diabetes mellitus, dyslipidemia, chronic kidney disease, and hyperuricemia as well as systolic blood pressure, fasting plasma glucose level, blood glycosylated hemoglobin (hemoglobin A_1c_) content, and serum concentrations of triglycerides, creatinine, and uric acid were greater, whereas serum concentrations of high density lipoprotein (HDL)–cholesterol and estimated glomerular filtration rate (eGFR) were lower, in subjects with CAD or MI than in controls.

**Table 1:** Characteristics of the 12,698 study subjects

Characteristic	Control	Coronary artery disease	*P*	Myocardial infarction	*P*
No. of subjects	9210	3488		2438	
Age (years)	58.8 ± 13.8	68.1 ± 13.0	<0.0001	67.0 ± 13.3	<0.0001
Sex (male/female, %)	50.4/49.6	71.5/28.5	<0.0001	74.7/25.3	<0.0001
Body mass index (kg/m^2^)	23.1 ± 3.5	23.9 ± 3.4	<0.0001	24.0 ± 3.4	<0.0001
Current or former smoker (%)	37.2	36.4	0.4742	38.7	0.2039
Hypertension (%)	42.4	78.1	<0.0001	76.7	<0.0001
Systolic blood pressure (mmHg)	125 ± 20	141 ± 26	<0.0001	140 ± 27	<0.0001
Diastolic blood pressure (mmHg)	75 ± 12	75 ± 15	0.7032	76 ± 16	0.1412
Diabetes mellitus (%)	14.5	51.3	<0.0001	53.1	<0.0001
Fasting plasma glucose (mmol/L)	5.80 ± 1.95	7.42 ± 3.26	<0.0001	7.54 ± 3.28	<0.0001
Blood hemoglobin A_1c_ (%)	5.70 ± 0.93	6.70 ± 1.67	<0.0001	6.76 ± 1.66	<0.0001
Dyslipidemia (%)	57.6	79.0	<0.0001	80.0	<0.0001
Serum triglycerides (mmol/L)	1.38 ± 0.96	1.63 ± 1.13	<0.0001	1.60 ± 1.07	<0.0001
Serum HDL-cholesterol (mmol/L)	1.62 ± 0.44	1.23 ± 0.38	<0.0001	1.20 ± 0.36	<0.0001
Serum LDL-cholesterol (mmol/L)	3.13 ± 0.80	3.12 ± 0.99	0.6627	3.14 ± 0.98	0.7934
Chronic kidney disease (%)	18.8	37.9	<0.0001	37.9	<0.0001
Serum creatinine (μmol/L)	72.9 ± 68.4	94.5 ± 111.8	<0.0001	94.2 ± 101.4	<0.0001
eGFR (mL min^−1^ 1.73 m^−2^)	74.0 ± 18.1	66.6 ± 24.8	<0.0001	67.0 ± 26.1	<0.0001
Hyperuricemia (%)	16.1	23.7	<0.0001	24.3	<0.0001
Serum uric acid (μmol/L)	323 ± 90	348 ± 103	<0.0001	353 ± 105	<0.0001

Quantitative data are means ± SD and were compared between subjects with coronary artery disease or myocardial infarction and control individuals with the unpaired Student's *t* test. Categorical data were compared between two groups with Fisher's exact test. Based on Bonferroni's correction, a *P* value of <0.0013 (0.05/38) was considered statistically significant. HDL, high density lipoprotein; LDL, low density lipoprotein; eGFR, estimated glomerular filtration rate.

### EWAS for CAD

We examined the relation of allele frequencies of 41,339 SNPs that passed quality control to CAD with the use of Fisher's exact test. A Manhattan plot for the EWAS of CAD is shown in Supplementary Figure 1A. After Bonferroni's correction, 126 SNPs were significantly (*P* < 1.21 × 10^−6^) associated with CAD (Supplementary Table 1). The genotype distributions of these SNPs were in Hardy-Weinberg equilibrium (*P* > 0.001) among controls (Supplementary Table 2).

### Multivariable logistic regression analysis of the relation of SNPs to CAD

The relation of the 126 identified SNPs to CAD was further examined by multivariable logistic regression analysis with adjustment for age, sex, and the prevalence of hypertension, diabetes mellitus, and dyslipidemia (Supplementary Table 3). Six SNPs were related (*P* < 0.01 in at least one genetic model) to CAD (Table 2), but none of these polymorphisms showed a significant [*P* < 9.92 × 10^−5^ (0.05/504)] association with this condition.

**Table 2:** Relation of single nucleotide polymorphisms (SNPs) to coronary artery disease as determined by multivariable logistic regression analysis

SNP		Dominant		Recessive		Additive 1		Additive 2	
		*P*	OR (95% CI)	*P*	OR (95% CI)	*P*	OR (95% CI)	*P*	OR (95% CI)
rs58098972	A/G	0.0064	1.16 (1.04–1.29)	0.2601		0.0118	1.15 (1.03–1.29)	0.1780	
rs77336780	C/G (A304G)	0.0355	0.90 (0.81–0.99)	0.0145	0.74 (0.57–0.94)	0.1381		0.0085	0.72 (0.55–0.92)
rs200121865	G/C (G149A)	0.0034	2.27 (1.32–3.86)	0.0682		0.0065	2.16 (1.25–3.69)	0.0679	
rs202069030	G/C (R51S)	0.0099	0.32 (0.09–0.78)	ND		0.0099	0.32 (0.09–0.78)	ND	
rs7188	T/G	0.5784		0.0009	0.76 (0.64–0.89)	0.6171		0.0025	0.77 (0.65–0.91)
rs2271395	A/G (T1587A)	0.0023	0.85 (0.77–0.94)	0.0918		0.0084	0.86 (0.77–0.96)	0.0052	0.82 (0.72–0.94)

Multivariable logistic regression analysis was performed with adjustment for age, sex, and the prevalence of hypertension, diabetes mellitus, and dyslipidemia. Based on Bonferroni's correction, a *P* value of <9.92 × 10^−5^ (0.05/504) was considered statistically significant. OR, odds ratio; CI, confidence interval; ND, not determined.

### EWAS for MI

We next examined the relation of allele frequencies of the 41,339 SNPs to MI with Fisher's exact test. A Manhattan plot for the EWAS of MI is shown in Supplementary Figure 1B. After Bonferroni's correction, 114 SNPs were significantly (*P* < 1.21 × 10^−6^) associated with MI (Supplementary Table 4). The genotype distributions of these SNPs were in Hardy-Weinberg equilibrium (*P* > 0.001) among subjects with MI and controls (Supplementary Table 5).

### Multivariable logistic regression analysis of the relation of SNPs to MI

The relation of the 114 identified SNPs to MI was further examined by multivariable logistic regression analysis with adjustment for age, sex, and the prevalence of hypertension, diabetes mellitus, and dyslipidemia (Supplementary Table 6). Nine SNPs were related (*P* < 0.01 in at least one genetic model) to MI (Table 3). Among these SNPs, rs188212047 [G/T (L212F)] of *STXBP2* (dominant and additive 1 models) was significantly [*P* < 1.10 × 10^−4^ (0.05/456)] associated with MI, with the minor *T* allele representing a risk factor for this condition.

**Table 3:** Relation of single nucleotide polymorphisms (SNPs) to myocardial infarction as determined by multivariable logistic regression analysis

SNP		Dominant		Recessive		Additive 1		Additive 2	
		*P*	OR (95% CI)	*P*	OR (95% CI)	*P*	OR (95% CI)	*P*	OR (95% CI)
rs202103723	C/A (P511Q)	0.0006	2.68 (1.54–4.58)	0.0448	>100 (ND)	0.0015	2.54 (1.44–4.37)	0.0443	>100 (ND)
rs188212047	G/T (L212F)	**4.84 × 10^−8^**	2.94 (2.02–4.24)	ND		**4.84 × 10^−8^**	2.94 (2.02–4.24)	ND	
rs1265110	G/A	0.0005	0.82 (0.74–0.92)	0.0719		0.0020	0.83 (0.74–0.94)	0.0114	0.77 (0.62–0.94)
rs138559558	G/A (R289C)	0.9473		0.0017	>100 (ND)	0.7886		0.0017	>100 (ND)
rs11007350	C/T	0.0067	0.86 (0.77–0.96)	0.0394	0.77 (0.60–0.99)	0.0270	0.88 (0.78–0.99)	0.0155	0.73 (0.57–0.94)
rs9258102	T/C	0.0010	1.25 (1.10–1.43)	0.7389		0.0010	1.26 (1.10–1.45)	0.6189	
rs200867550	C/T (V874I)	0.0083	0.13 (0.01–0.65)	ND		0.0083	0.13 (0.01–0.65)	ND	
rs9293471	A/G	0.0344	0.89 (0.80–0.99)	0.0244	0.80 (0.66–0.97)	0.1373		0.0100	0.77 (0.63–0.94)
rs439121	G/T	0.1481		0.0060	1.25 (1.07–1.46)	0.5685		0.0056	1.27 (1.07–1.51)

Multivariable logistic regression analysis was performed with adjustment for age, sex, and the prevalence of hypertension, diabetes mellitus, and dyslipidemia. Based on Bonferroni's correction, *P* values of <1.10 × 10^−4^ (0.05/456) were considered statistically significant and are shown in bold. OR, odds ratio; CI, confidence interval; ND, not determined.

### Association of rs188212047 with MI in men or women

We examined the relation of rs188212047 of *STXBP2* to MI for men (1821 subjects with MI, 4642 controls) or for women (617 subjects with MI, 4568 controls) separately. Multivariable logistic regression analysis with adjustment for age and the prevalence of hypertension, diabetes mellitus, and dyslipidemia revealed that rs188212047 was significantly associated with MI both for men (*P* = 7.66 × 10^−6^; odds ratio, 2.73; 95% confidence interval, 1.77–4.19) and for women (*P* = 0.0011; odds ratio, 3.77; 95% confidence interval, 1.76–7.54).

### Relation of SNPs to intermediate phenotypes of MI

We examined the relation of rs188212047 of *STXBP2* and the other eight SNPs found to be related (*P* < 0.01) to MI to intermediate phenotypes of MI—including hypertension, diabetes mellitus, hypertriglyceridemia, hypo–HDL-cholesterolemia, hyper–low density lipoprotein (LDL)–cholesterolemia, chronic kidney disease, obesity, and hyperuricemia—with the use of Fisher's exact test or Pearson's chi-square test. No significant association was apparent between any of these SNPs and intermediate phenotypes (Supplementary Table 7).

### Relation of chromosomal loci, genes, and SNPs identified in the present study to phenotypes previously examined in GWASs

We examined the 15 loci, genes, and SNPs identified in the present study to phenotypes previously examined by GWASs available in public databases [GWAS Catalog (http://www.ebi.ac.uk/gwas) and GWAS Central (http://www.gwascentral.org/browser)]. None of these loci, genes, or SNPs was found to be associated with CAD or MI in previous GWASs (Supplementary Table 8).

## DISCUSSION

We have now shown that rs188212047 [G/T (L212F)] of *STXBP2* was significantly associated with MI in Japanese, with the minor *T* allele representing a risk factor for this condition. This SNP was associated with MI both in men and in women. We also identified an additional six and eight SNPs as candidate loci for CAD and MI, respectively.

The syntaxin binding protein 2 gene (*STXBP2*) is located at chromosomal region 19p13.2 (NCBI Gene,https://www.ncbi.nlm.nih.gov/gene) and is expressed in various tissues and organs including vascular smooth muscle (The Human Protein Atlas,http://www.proteinatlas.org). STXBP2 is a cytosolic protein that interacts with syntaxin-1A, -1B, -2, and -3 [17] and functions in intracellular trafficking and control of soluble NSF attachment protein receptor (SNARE) complex assembly [17]. It plays a role in exocytosis from mast cells and neutrophils as well as in the release of cytotoxic granules by natural killer (NK) cells [17–19]. Mutations in *STXBP2* are associated with familial hemophagocytic lymphohistiocytosis (FHL), a life-threatening hyperinflammatory syndrome that results from an uncontrolled and ineffective immune response due to dysfunction of cytotoxic T lymphocytes (CTLs) and NK cells [18–20]. The defect in cytotoxic activity prevents efficient removal of antigens and down-regulation of the immune response, resulting in sustained activation and proliferation of CTLs and NK cells [21]. The persistently activated CTLs and NK cells produce large amounts of cytokines and thereby give rise to the activation of histiocytes (macrophages and dendritic cells). These latter cells in turn migrate to sites occupied by CTLs and NK cells and further promote their activation, tissue infiltration, and secretion of inflammatory cytokines [22]. Organ infiltration by activated lymphocytes and histiocytes and the hypercytokinemia generated by these cells thus play a key role in the pathogenesis of FHL [23].

We have now shown that rs188212047 [G/T (L212F)] of *STXBP2* was significantly associated with MI, with the minor *T* allele representing a risk factor this condition. Given that vascular inflammation is an important contributor to the development of coronary atherosclerosis and thrombosis [24, 25] and that *STXBP2* is implicated in the regulation of inflammation and the immune response [18–23], the association of rs188212047 [G/T (L212F)] of *STXBP2* with MI might be attributable to the effect of this gene on vascular inflammation.

In previous meta-analyses of GWASs for CAD, the MAF of SNPs ranged from 3% to 48% and the odds ratio from 0.67 to 1.23 [11, 13]. In our study, we identified *STXBP2* as a novel susceptibility locus for MI, with the allele odds ratio and MAF of rs188212047 being 2.51 and 0.8%, respectively. This SNP is thus a low-frequency variant with a moderate to large effect size. We also detected an additional 14 candidate loci for CAD (rs58098972 of *WDR66*, rs77336780 of *OR51I1*, rs200121865 of *KLHDC2*, rs202069030 of *DUS2*, rs7188 of *KANK2*, and rs2271395 of *N4BP2*) or for MI (rs202103723 of *CWH43*, rs1265110 of *CCHCR1*, rs138559558 of *UBXN11*, rs11007350 at 10p12.2, rs9258102 at 6p21.3, rs200867550 of *ROR2*, rs9293471 at 5q14, and rs439121 at 6p21.3). Although rs58098972 (allele odds ratio, 1.08; MAF, 14.5%), rs77336780 (0.94; 20.6%), rs7188 (0.93; 32.3%), rs2271395 (0.91; 45.9%), rs1265110 (0.87; 30.2%), rs11007350 (0.90; 23.8%), rs9258102 (1.20; 10.8%), rs9293471 (0.93; 30.4%), and rs439121 (1.10; 35.0%) were common variants [rs138559558 (1.08; 1.2%) was low-frequency variant] with small effect sizes, rs200121865 (2.06; 0.3%), rs202069030 (0.10; 0.4%), rs202103723 (2.63; 0.3%), and rs200867550 (0.22; 0.2%) were rare variants with moderate to large effect sizes. Previous GWASs [3–6] and a meta-analysis of GWASs [12] have implicated chromosome 9p21.3 (*CDKN2B-AS1*) in predisposition to CAD or MI in Caucasian populations. None of the SNPs at this locus (rs10757274, rs2383206, rs2383207, rs10757278, rs1333049, rs1333045) were included in the human exome arrays we used.

There are several limitations to our study: (i) Given that the results were not replicated, their validation will be necessary in other independent subject panels or in other ethnic groups. (ii) It is possible that rs188212047 of *STXBP2* is in linkage disequilibrium with other polymorphisms in the same gene or in other nearby genes that are actually responsible for the development of MI. (iii) The functional relevance of rs188212047 to the pathogenesis of MI remains to be elucidated.

In conclusion, rs188212047 of *STXBP2* may be a susceptibility locus for MI in Japanese. Determination of genotypes for this SNP may prove informative for assessment of the genetic risk for MI in Japanese.

## MATERIALS AND METHODS

### Study subjects

A total of 12,698 Japanese (3488 subjects with CAD, including 2438 with MI; 9210 controls) was examined. The subjects were recruited from individuals either who visited outpatient clinics of or were admitted to participating hospitals (Gifu Prefectural Tajimi Hospital, Tajimi; Gifu Prefectural General Medical Center, Gifu; Japanese Red Cross Nagoya First Hospital, Nagoya; Inabe General Hospital, Inabe; Hirosaki University Hospital and Hirosaki Stroke Center, Hirosaki, Japan) because of various symptoms or for an annual health checkup between 2002 and 2014; who were community-dwelling individuals recruited to a population-based cohort study in Inabe between 2010 and 2014 or in Tokyo or Kusatsu between 2011 and 2015; or who underwent autopsy at the Tokyo Metropolitan Geriatric Hospital from 1995 to 2012.

The diagnosis of CAD was based on the detection of stenosis of >50% in any major coronary artery or in the left main trunk by coronary angiography. The diagnosis of MI was based on typical electrocardiographic changes and on increases both in the serum activity of creatine kinase (MB isozyme) and in the serum concentration of troponin T. The diagnosis was confirmed by identification of the responsible stenosis in any of the major coronary arteries or in the left main trunk by coronary angiography. In autopsy cases, the diagnosis was pathologically confirmed by the detection of myocardial necrosis and the responsible stenosis in any of the major coronary arteries or in the left main trunk. The control individuals had no history of MI, CAD, aortic aneurysm, or peripheral artery disease; of ischemic or hemorrhagic stroke; or of other atherosclerotic, thrombotic, embolic, or hemorrhagic disorders. Autopsy cases were excluded from controls. Although some control individuals had conventional risk factors for CAD—including hypertension (systolic blood pressure of ≥140 mmHg, diastolic blood pressure of ≥90 mmHg, or taking of anti-hypertensive medication), diabetes mellitus (fasting blood glucose concentration of ≥6.93 mmol/L, blood glycosylated hemoglobin content of ≥6.5%, or taking of antidiabetes medication), dyslipidemia (serum triglyceride concentration of ≥1.65 mmol/L, serum HDL-cholesterol concentration of <1.04 mmol/L, serum LDL-cholesterol concentration of ≥3.64 mmol/L, or taking of anti-dyslipidemic medication), chronic kidney disease [eGFR of <60 mL min^−1^ 1.73 m^−2^, where eGFR (mL min^−1^ 1.73 m^−2^) = 194 × (age in years)^−0.287^ × (serum creatinine in mg/dL)^−1.094^ (× 0.739 if female)], and hyperuricemia (serum concentration of uric acid of ≥416 μmol/L)—they did not have cardiovascular complications.

The study protocol complied with the Declaration of Helsinki and was approved by the Committees on the Ethics of Human Research of Mie University Graduate School of Medicine, Hirosaki University Graduate School of Medicine, Tokyo Metropolitan Institute of Gerontology, and participating hospitals. Written informed consent was obtained from all subjects or families of the deceased subjects.

### EWAS

Venous blood (5 or 7 mL) was collected into tubes containing 50 mmol/L ethylenediaminetetraacetic acid (disodium salt), peripheral blood leukocytes were isolated, and genomic DNA was extracted from these cells with the use of a DNA extraction kit (Genomix supplied by Talent, Trieste, Italy; or SMITEST EX-R&D supplied by Medical & Biological Laboratories, Nagoya, Japan) or by standard protocols based on phenol-chloroform extraction and spin columns. In autopsy cases, genomic DNA was extracted from kidneys. The EWASs for CAD or MI were performed with 3488 CAD patients or 2438 MI patients and with 9210 controls with the use of a HumanExome-12 v1.1 or v1.2 DNA Analysis BeadChip or Infinium Exome-24 v1.0 BeadChip (Illumina, San Diego, CA, USA). These exome arrays include putative functional exonic variants selected from >12,000 individual exome and whole-genome sequences. The exonic content consists of ~244,000 SNPs representing diverse populations, including European, African, Chinese, and Hispanic individuals [26]. SNPs contained in only one of the exome arrays (~3.6% of all SNPs) were excluded from analysis. We performed quality control [27] as follows: (i) Genotyping data with a call rate of <97% were discarded, with the mean call rate for the remaining data being 99.9%. (ii) Gender specification was checked for each sample, and those for which gender phenotype in the clinical records was inconsistent with genetic sex were discarded. (iii) Cryptic relatedness and duplicate samples were checked by calculation of identity by descent; all pairs of DNA samples showing an identity by descent of >0.1875 were inspected and one sample from each pair was excluded. (iv) The frequency of heterozygosity for SNPs was calculated for all samples, and those with extremely low or high heterozygosity (>3 standard deviations from the mean) were discarded. (v) SNPs in sex chromosomes or mitochondrial DNA were excluded from the analysis, as were nonpolymorphic SNPs or SNPs with a MAF of <0.1%. (vi) SNPs whose genotype distributions deviated significantly (*P* < 0.001) from Hardy-Weinberg equilibrium in controls were discarded. (vii) Genotype data in each EWAS were examined for population stratification by principal components analysis [28], and population outliers were excluded from the analysis. A two-dimensional display of the samples examined by such analysis is presented in Supplementary Figure 2. A total of 41,339 SNPs passed quality control and was subjected to subsequent analysis.

### Statistical analysis

For analysis of the characteristics of the study subjects, quantitative data were compared between patients with CAD or MI and controls with the unpaired Student's *t* test. Categorical data were compared between the two groups with Fisher's exact test. Allele frequencies were estimated by the gene counting method, and Fisher's exact test was applied to identify departure from Hardy-Weinberg equilibrium. We examined the relation of allele frequencies of each SNP to CAD or MI with Fisher's exact test. To compensate for multiple comparisons of genotypes with CAD or MI, we applied Bonferroni's correction for statistical significance of association. Given that 41,339 SNPs that passed quality control were examined, a *P* value of <1.21 × 10^−6^ (0.05/41,339) was considered statistically significant for each EWAS. Quantile-quantile plots for *P* values of allele frequencies in the EWAS for CAD or MI are shown in Supplementary Figure 3. The inflation factor (λ) was 1.06 for CAD and 1.16 for MI. Multivariable logistic regression analysis was performed with CAD or MI as a dependent variable and independent variables including age, sex (0, woman; 1, man), the prevalence of hypertension, diabetes mellitus, and dyslipidemia (0, no history of these conditions; 1, positive history), as well as genotype of each SNP. Genotypes of each SNP were assessed according to dominant [0, *AA*; 1, *AB* + *BB* (*A*, major allele; *B*, minor allele)], recessive (0, *AA* + *AB*; 1, *BB*), and additive genetic models, and the *P* value, odds ratio, and 95% confidence interval were calculated. Additive models comprised additive 1 (0, *AA*; 1, *AB*; 0, *BB*) and additive 2 (0, *AA*; 0, *AB*; 1, *BB*) scenarios, which were analyzed simultaneously with a single statistical model. The relation of genotypes of SNPs to intermediate phenotypes was examined with Fisher's exact test (2 × 2) or Pearson's chi-square test (2 × 3). Bonferroni's correction was also applied to other statistical analysis as indicated. Statistical tests were performed with JMP Genomics version 6.0 software (SAS Institute, Cary, NC).

## SUPPLEMENTARY MATERIALS FIGURES AND TABLES




