# Supplementary material for: Identification of STXBP2 as a novel susceptibility locus for myocardial infarction in Japanese individuals by an exome-wide association study
Source: Oncotarget. 2017 Mar 23;8(20):33527–35. doi: 10.18632/oncotarget.16536 (PMC5464887; doi:10.18632/oncotarget.16536)
Supplement: Supplementary file 1 [file oncotarget-08-33527-s001.pdf]

Identification of *STXBP2* as a novel susceptibility locus for myocardial infarction in Japanese individuals by an exome-wide association study

SUPPLEMENTARY MATERIALS

SUPPLEMENTARY FIGURES AND TABLES

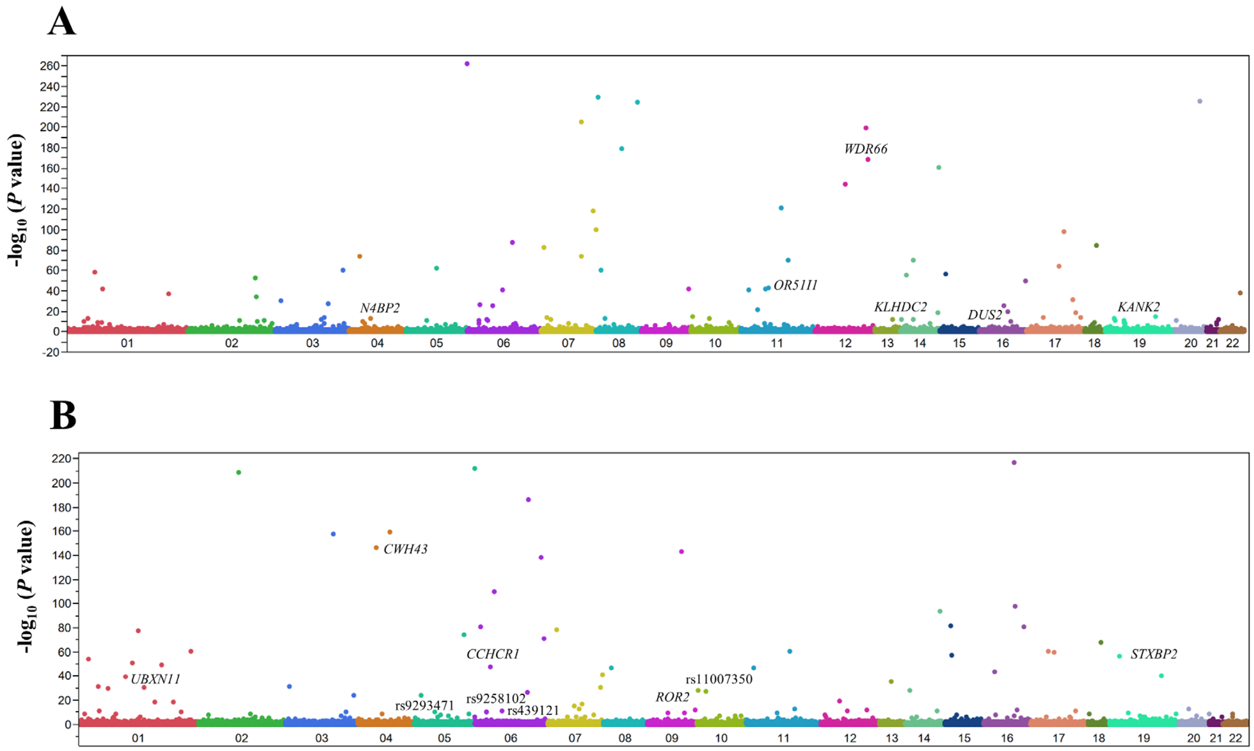

Supplementary Figure 1: Manhattan plots for *P* values of allele frequencies in the EWAS of CAD **(A)** or MI **(B)**. The *P* values are shown as $-\log_{10}(P)$ on the *y*-axis with respect to the physical chromosomal position of the corresponding SNP on the *x*-axis. SNPs or the corresponding genes identified in the present study are indicated.

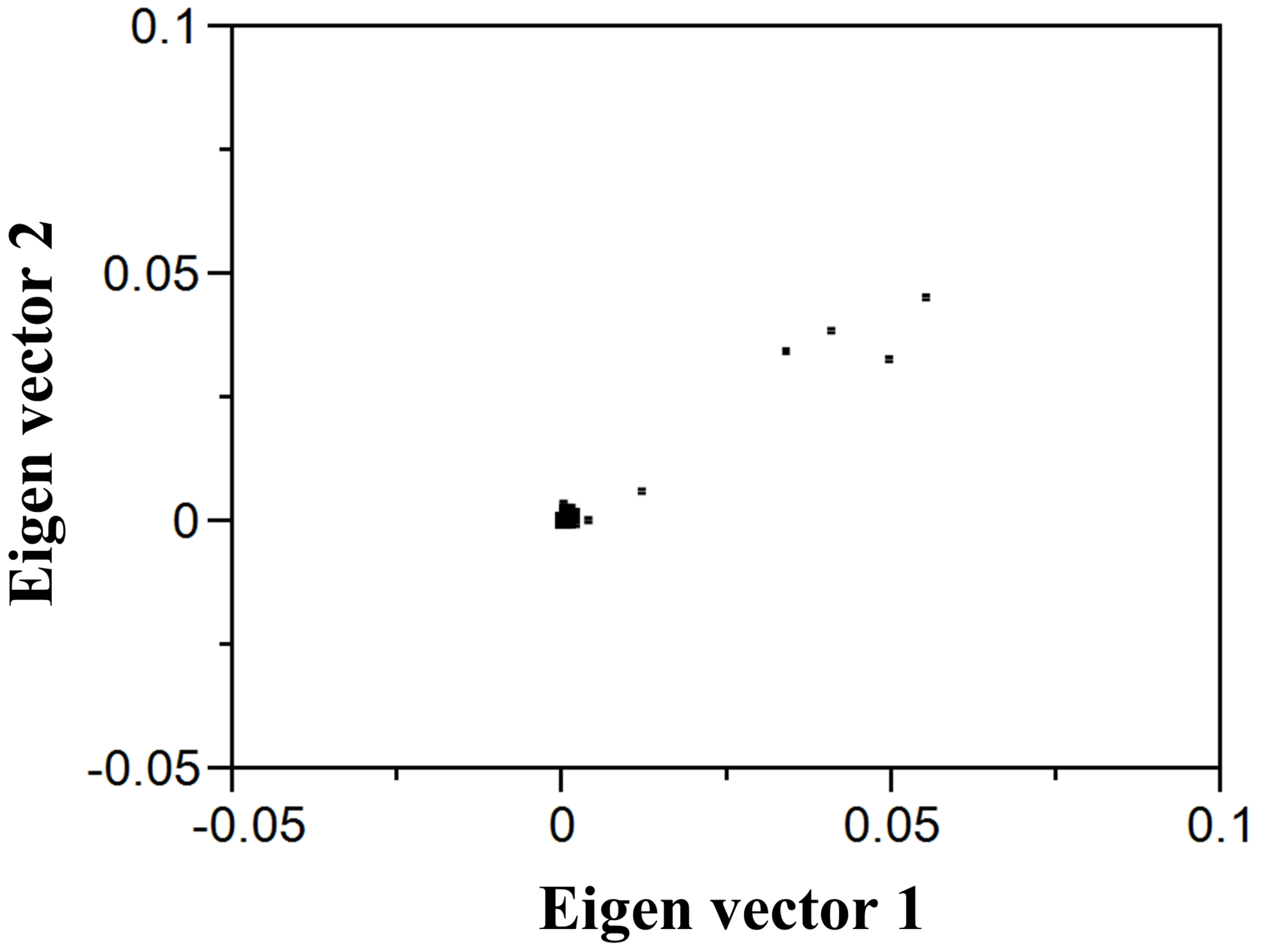

Supplementary Figure 2: Distribution of samples examined by principal components analysis for population stratification in the EWAS for CAD. The samples enrolled in the EWAS examined by principal components analysis with the EIGENSTRAT method are plotted according to the first (horizontal axis) and second (vertical axis) principal components.

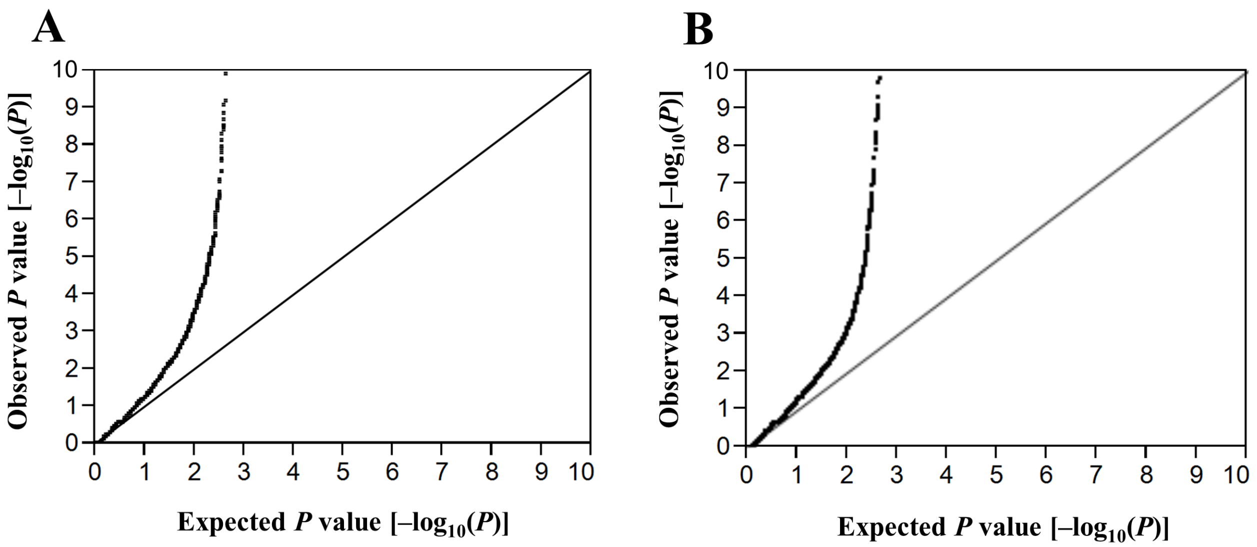

Supplementary Figure 3: Quantile-quantile plots for *P* values of allele frequencies in the EWASs of CAD **(A)** or MI **(B)**. The observed *P* values (*y*-axis) were compared with the expected *P* values (*x*-axis) under the null hypothesis, with the values being plotted as $-\log_{10}(P)$.

Supplementary Table 1: The 126 single nucleotide polymorphisms (SNPs) significantly ($P < 1.21 \times 10^{-6}$) associated with coronary artery disease in the exome-wide association study

See Supplementary File 1

Supplementary Table 2: Genotype distributions for single nucleotide polymorphisms (SNPs) significantly associated with coronary artery disease in the exome-wide association study

See Supplementary File 1

Supplementary Table 3: Relation of single nucleotide polymorphisms (SNPs) to coronary artery disease as determined by multivariable logistic regression analysis

See Supplementary File 1

Supplementary Table 4: The 114 single nucleotide polymorphisms (SNPs) significantly ($P < 1.21 \times 10^{-6}$) associated with myocardial infarction in the exome-wide association study

See Supplementary File 1

Supplementary Table 5: Genotype distributions for single nucleotide polymorphisms (SNPs) significantly associated with myocardial infarction in the exome-wide association study

See Supplementary File 1

Supplementary Table 6: Relation of single nucleotide polymorphisms (SNPs) to myocardial infarction as determined by multivariable logistic regression analysis

See Supplementary File 1

Supplementary Table 7: Relation of single nucleotide polymorphisms (SNPs) to intermediate phenotypes of myocardial infarction

SNP		Hypertension	DM	Hyper-TG	Hypo-HDL	Hyper-LDL	CKD	Obesity	HU
rs202103723	C/A (P511Q)	0.5124	0.5163	0.1918	0.0020	0.7466	0.7494	0.0900	0.0819
rs188212047	G/T (L212F)	0.0757	0.2205	0.9341	0.5152	0.8698	0.5681	0.7313	0.5970
rs1265110	G/A	0.4085	0.6213	0.8101	0.1721	0.0876	0.6498	0.5902	0.7675
rs138559558	G/A (R289C)	0.5494	0.7463	0.5219	0.1377	0.4961	0.5753	0.1021	0.3497
rs11007350	C/T	0.6215	0.9710	0.8261	0.0927	0.1360	0.9195	0.8144	0.1103
rs9258102	T/C	0.6187	0.4197	0.9346	0.7600	0.0049	0.6706	0.9573	0.3608
rs200867550	C/T (V874I)	0.7394	0.4095	0.8579	0.6446	0.5975	0.8403	0.8563	0.1131
rs9293471	A/G	0.9405	0.8981	0.7197	0.5469	0.8744	0.8615	0.6902	0.1117
rs439121	G/T	0.3721	0.8296	0.5487	0.2394	0.3338	0.1405	0.3677	0.0504

Data are *P* values. The relation of genotypes of each SNP to intermediate phenotypes was examined with Fisher's exact test (2 × 2) or Pearson's chi-square test (2 × 3). DM, diabetes mellitus; hyper-TG, hypertriglyceridemia; hypo-HDL, hypo–HDL-cholesterolemia; hyper-LDL, hyper–LDL-cholesterolemia; CKD, chronic kidney disease; HU, hyperuricemia. Based on Bonferroni's correction, a *P* value of $<6.94 \times 10^{-4}$ (0.05/72) was considered statistically significant.

Supplementary Table 8: Relation of chromosomal loci, genes, and single nucleotide polymorphisms (SNPs) identified in the present study to phenotypes previously examined in genome-wide association studies

See Supplementary File 1
