# Supplementary material for: Identification of STXBP2 as a novel susceptibility locus for myocardial infarction in Japanese individuals by an exome-wide association study
Source: Oncotarget. 2017 Mar 23;8(20):33527–35. doi: 10.18632/oncotarget.16536 (PMC5464887; doi:10.18632/oncotarget.16536)
Supplement: Supplementary file 2 [file oncotarget-08-33527-s002.docx]

**Supplementary Table 1.** The 126 single nucleotide polymorphisms (SNPs) significantly (*P* < 1.21 × 10^–6^) associated with coronary artery disease in the exome-wide association study.

___________________________________________________________________________________

Gene dbSNP Nucleotide Chromosome: MAF *P* (allele) Allele

(amino acid) position (%) OR

substitution^a^

___________________________________________________________________________________

*GMDS* rs9378305 C/T 6: 1703056 41.4 5.31 × 10^–263^ 1.01

*XKR5* rs2741098 C/T (V69M) 8: 6832754 43.7 5.38 × 10^–230^ 1.00

*NFATC2* rs12479626 T/C (H426R) 20: 51475656 4.7 4.41 × 10^–226^ 1.10

*ZC3H3* rs3750208 G/A (R168W) 8: 143538865 11.3 6.49 × 10^–225^ 0.96

*LMOD2* rs7809453 G/A 7: 123661886 42.8 3.93 × 10^–206^ 1.01

rs7299095 G/A 12: 118458499 40.3 1.57 × 10^–200^ 0.98

*NBN* rs192236678 G/T (F521L) 8: 89953280 0.4 1.14 × 10^–179^ 0.97

*WDR66* rs58098972 A/G 12: 121921491 14.5 1.99 × 10^–169^ 1.08

*AHNAK2* rs181990876 C/T (G450S) 14: 104954103 0.4 5.18 × 10^–161^ 0.66

rs11171747 T/G 12: 56124624 32.9 2.72 × 10^–145^ 0.99

*CTSW* rs115991011 C/A (A361D) 11: 65883569 0.5 2.20 × 10^–121^ 1.16

rs28505524 T/G 7: 150749939 25.4 4.72 × 10^–119^ 1.02

*NOM1* rs2302445 G/A (R779H) 7: 156969124 27.7 2.70 × 10^–100^ 1.00

rs807122 T/C 17: 53157701 21.0 3.06 × 10^–98^ 1.03

*GABRR2* rs138360169 T/C (N457S) 6: 89257698 0.1 8.78 × 10^–88^ 0.28

*TCEB3B* rs2010834 A/C (F254C) 18: 47034504 24.5 1.94 × 10^–85^ 0.98

*USP42* rs7784072 G/C 7: 6154635 7.4 1.52 × 10^–83^ 0.96

*CCDC149* rs12511068 C/T (V7I) 4: 24895036 12.4 1.31 × 10^–74^ 1.11

*ASB15* rs4731112 C/G (A357G) 7: 123629064 15.9 2.46 × 10^–74^ 1.04

*MIS18BP1* rs145716748 A/G (S729P) 14: 45224402 1.9 9.71 × 10^–71^ 1.08

*LIPT2* rs586088 A/T (T190S) 11: 74492263 31.5 1.09 × 10^–70^ 1.01

*GPATCH8* rs185067598 A/G (L277P) 17: 44401013 0.3 4.60 × 10^–65^ 0.85

*SLCO6A1* rs17150488 T/C (K381R) 5: 102438751 0.6 2.76 × 10^–63^ 1.06

*SENP2* rs6762208 C/A (T301K) 3: 185613377 38.6 2.15 × 10^–61^ 1.02

rs7828656 A/C 8: 11645425 44.8 4.37 × 10^–61^ 1.04

rs6704425 C/T 1: 38410319 22.6 3.45 × 10^–59^ 0.96

*C15orf57* rs3803354 T/C 15: 40564790 8.9 1.78 × 10^–57^ 0.98

*SLC7A8* rs2236133 A/G 14: 23159618 29.9 3.04 × 10^–56^ 0.97

*PARD3B* rs2216317 G/A 2: 204640625 19.4 7.71 × 10^–53^ 0.99

*TUBB3* rs2302898 A/G 16: 89932386 23.5 9.36 × 10^–51^ 0.94

*OR4X2* rs7120775 C/G (Y27*) 11: 48245184 16.9 1.50 × 10^–43^ 0.97

ABCA2 rs2271862 A/G 9: 137011907 19.2 1.12 × 10^–42^ 0.95

rs12807582 G/T 11: 39306013 48.0 1.14 × 10^–42^ 0.99

rs6695567 A/G 1: 53163913 43.6 2.56 × 10^–42^ 1.02

*OR51I1* rs77336780 C/G (A304G) 11: 5440604 20.6 4.49 × 10^–42^ 0.94

*PKHD1* rs141384205 G/A (R559W) 6: 52056716 1.3 6.89 × 10^–42^ 1.47

*RIBC2* rs2142662 G/A 22: 45426055 14.2 9.10 × 10^–39^ 0.89

*CENPF* rs79923436 G/A (S2302N) 1: 214646475 1.9 9.66 × 10^–38^ 0.95

*GPR1* rs34685097 G/A (R236*) 2: 206176542 1.2 1.26 × 10^–34^ 0.88

*TTYH2* rs9899862 C/A (D423E) 17: 74253090 6.3 1.49 × 10^–31^ 0.99

*TMEM43* rs2340917 C/T (T179M) 3: 14133762 45.9 2.09 × 10^–31^ 1.01

*AMOTL2* rs1353776 G/C (E729D) 3: 134358628 5.0 3.20 × 10^–28^ 1.09

*ATAT1* rs34315095 C/G 6: 30625708 4.7 1.02 × 10^–26^ 0.94

*BRPF3* rs3748045 C/G 6: 36230800 31.0 4.52 × 10^–26^ 0.92

rs1528601 C/G 16: 51064516 29.6 7.24 × 10^–26^ 0.99

*USP47* rs138329346 C/T (H313Y) 11: 11920417 2.4 7.34 × 10^–22^ 0.93

rs16963698 A/G 16: 61624110 45.4 1.72 × 10^–20^ 1.03

*TRMT61A* rs200587171 C/T 14: 103534551 0.9 1.48 × 10^–19^ 0.91

*ITGB4* rs871443 C/T (P1779L) 17: 75757422 35.2 1.57 × 10^–19^ 1.00

*FAM208B* rs2254067 G/T (G499C) 10: 5739665 22.4 1.57 × 10^–15^ 1.07

*LMTK3* rs140955674 G/A (T1426I) 19: 48491442 6.2 2.12 × 10^–15^ 0.95

*RAI1* rs200517965 A/T (D635V) 17: 17794852 0.3 1.03 × 10^–14^ 1.03

*GATA2* rs78245253 G/C (A250P) 3: 128485850 4.6 1.16 × 10^–14^ 0.94

*LOC101927630* rs10499504 A/G 7: 17521959 9.0 2.23 × 10^–14^ 0.99

*RNF213* rs10782008 G/A (V1195M) 17: 80332071 33.7 3.90 × 10^–14^ 0.96

*GPR108* rs117917124 C/T (V289I) 19: 6733055 1.4 6.05 × 10^–14^ 1.07

*UBA6* rs10010188 C/T (A224T) 4: 67668674 14.9 1.29 × 10^–13^ 0.97

rs2588941 C/T 10: 61829153 47.5 2.43 × 10^–13^ 1.03

*ZNF683* rs10794531 C/T (R53H) 1: 26367754 37.7 2.57 × 10^–13^ 1.04

*HR* rs12675375 C/T (G337D) 8: 22127432 43.4 3.19 × 10^–13^ 1.03

rs265654 A/G 13: 83152299 45.8 8.51 × 10^–13^ 0.98

rs1959607 T/C 14: 19984301 1.5 1.12 × 10^–12^ 1.01

rs6923504 C/G 6: 32460409 23.7 1.41 × 10^–12^ 1.05

*FAM221A* rs35928055 A/G (S240G) 7: 23698272 12.4 2.01 × 10^–12^ 1.03

*SPATC1L* rs113710653 C/T (E231K) 21: 46161921 1.9 2.38 × 10^–12^ 2.63

*PARP14* rs13093808 C/A (A561E) 3: 122700236 15.8 3.25 × 10^–12^ 0.98

*KLHDC2* rs200121865 G/C (G149A) 14: 49777933 0.3 3.48 × 10^–12^ 2.06

*PEX11G* rs2303146 A/C (C91W) 19: 7482188 45.3 4.30 × 10^–12^ 1.03

*GABBR1* rs3828923 G/A 6: 29622938 6.6 4.52 × 10^–12^ 0.88

rs1233397 C/T 6: 29577938 47.6 5.91 × 10^–12^ 1.11

*SPC24* rs74491133 C/T 19: 11147894 1.3 9.99 × 10^–12^ 1.10

*SCN7A* rs6738031 A/C (I958M) 2: 166423412 29.6 1.43 × 10^–11^ 1.02

*NYAP2* rs3748993 C/A (P586T) 2: 225627054 19.5 1.52 × 10^–11^ 1.06

*HMGCR* rs12654264 T/A 5: 75352778 48.5 1.99 × 10^–11^ 1.02

*TBC1D20* rs36088178 T/C (N79S) 20: 447909 5.1 2.54 × 10^–11^ 1.08

*HLA-DQA2* rs2395253 G/A 6: 32747383 1.7 2.77 × 10^–11^ 1.12

*TLR10* rs11466651 C/T (V298I) 4: 38774699 10.4 4.39 × 10^–11^ 1.03

rs2501279 C/T 1: 22041849 29.5 5.93 × 10^–11^ 0.93

*DUS2* rs202069030 G/C (R51S) 16: 68023050 0.4 6.56 × 10^–11^ 0.10

*MAP2* rs2271251 C/G (A82G) 2: 209653415 0.7 1.27 × 10^–10^ 1.60

*CFAP57* rs663824 A/G (N241D) 1: 43183837 23.9 7.16 × 10^–10^ 0.98

*TRABD2B* rs147317864 C/T (A262T) 1: 47801502 0.2 8.84 × 10^–10^ ND

*KANK2* rs7188 T/G 19: 11164463 32.3 9.16 × 10^–10^ 0.93

*GSTO1* rs201522765 C/T (P151L) 10: 104266154 0.3 1.49 × 10^–9^ 1.13

*MOCOS* rs1057251 T/C (V867A) 18: 36268618 3.1 1.50 × 10^–9^ 0.94

*C21orf33* rs2838497 C/G (L217V) 21: 44144884 13.8 2.08 × 10^–9^ 1.02

*ADIPOQ* rs6773957 A/G 3: 186855916 42.9 3.05 × 10^–9^ 0.96

LOC101927123 rs12185961 G/A 3: 127361625 24.5 3.29 × 10^–9^ 1.04

*DOCK6* rs8409 G/A 19: 11208815 45.5 4.17 × 10^–9^ 1.01

*ZNF804B* rs80006813 A/C (K589Q) 7: 89334747 0.2 5.35 × 10^–9^ 0.47

*RAG1* rs3740955 G/A (H249R) 11: 36574050 23.5 8.12 × 10^–9^ 1.06

*SCLY* rs3210400 G/A (A183T) 2: 238081747 27.0 1.09 × 10^–8^ 0.95

*LOC554223* rs1611196 T/C 6: 29790218 20.7 1.29 × 10^–8^ 1.23

*GALC* rs74887188 T/C (I282V) 14: 87965625 11.1 1.68 × 10^–8^ 0.92

*N4BP2* rs2271395 A/G (T1587A) 4: 40137056 45.9 2.46 × 10^–8^ 0.91

*SIM1* rs143803280 G/A 6: 100390370 0.2 2.64 × 10^–8^ 1.59

*IMPDH2* rs61729488 T/C (N771S) 3: 49030471 1.7 4.96 × 10^–8^ 0.96

*DSG4* rs36101975 C/T 18: 31376941 11.4 5.17 × 10^–8^ 0.99

*OR8K1* rs75289680 T/G (V45G) 11: 56346172 1.5 5.42 × 10^–8^ 0.87

*SLC23A1* rs33972313 C/T (V264M) 5: 139379813 0.4 8.80 × 10^–8^ 0.71

*IL12RB2* rs78198420 A/T (N271Y) 1: 67330663 1.4 1.02 × 10^–7^ 0.82

*SVEP1* rs3739451 A/T (I3161F) 9: 110404512 8.5 1.81 × 10^–7^ 1.00

*DSTYK* rs148815814 C/T (R592Q) 1: 205162079 0.2 1.84 × 10^–7^ 1.01

*IRGM* rs72553867 C/A (T94K) 5: 150848404 15.4 1.86 × 10^–7^ 1.04

*TRAPPC10* rs192670611 C/T (R915C) 21: 44087902 0.2 1.90 × 10^–7^ 1.42

*ALDH3B1* rs308341 G/A 11: 68027831 21.2 2.08 × 10^–7^ 0.98

rs806276 A/G 6: 90497632 20.1 2.34 × 10^–7^ 1.01

rs10100485 G/A 8: 51116680 37.9 2.59 × 10^–7^ 1.10

*CHD1* rs201752702 T/C (D133G) 5: 98902939 0.1 2.91 × 10^–7^ 1.23

*SLC9C1* rs28516377 C/T (G826S) 3: 112199368 30.2 3.19 × 10^–7^ 1.02

*AP1G2* rs201586390 C/T (R458H) 14: 23563417 1.1 3.43 × 10^–7^ 1.01

*LINC00536* rs799889 C/A 8: 116238657 41.9 3.87 × 10^–7^ 0.97

*NLGN1* rs118079207 G/A (R716H) 3: 174280978 0.7 4.38 × 10^–7^ 1.26

*KIAA1549* rs2251220 G/A (S849L) 7: 138917080 17.8 4.90 × 10^–7^ 0.98

*RELN* rs362726 T/C 7: 103566787 46.1 4.93 × 10^–7^ 1.02

*POPDC3* rs11962089 A/G 6: 105164345 4.8 5.27 × 10^–7^ 1.06

*CHRNB1* rs201776800 T/C (M465T) 17: 7456611 4.8 5.67 × 10^–7^ 1.09

*EP400* rs117463303 G/A 12: 132006275 1.9 5.91 × 10^–7^ 0.87

*HDAC9* rs801524 A/G 7: 18667295 40.2 6.42 × 10^–7^ 0.96

*CEACAM21* rs714106 A/C (T121N) 19: 41577497 46.1 6.62 × 10^–7^ 1.02

*NEPRO* rs3732813 T/C (T406A) 3: 113005814 23.9 8.04 × 10^–7^ 0.95

*SPICE1* rs57006145 T/G (T824P) 3: 113446633 7.2 8.29 × 10^–7^ 1.12

*ARAP1* rs141567247 C/T (G295R) 11: 72712335 1.1 8.65 × 10^–7^ 1.28

*TEX261* rs151285112 T/C (T38A) 2: 70993734 2.1 8.85 × 10^–7^ 0.86

*C10orf71* rs45554335 A/C (D461A) 10: 49323927 38.8 8.88 × 10^–7^ 1.09

*APOL5* rs2076671 T/C (M272T) 22: 35726883 43.8 1.01 × 10^–6^ 1.00

___________________________________________________________________________________

Allele frequencies were analyzed with Fisher’s exact test. ^a^Major allele/minor allele. MAF, minor allele frequency; OR, odds ratio; ND, not determined.

**Supplementary Table 2.** Genotype distributions for single nucleotide polymorphisms (SNPs) significantly associated with coronary artery disease in the exome-wide association study.

____________________________________________________________________________________________________________

SNP Coronary artery disease H-W *P* Control H-W *P*

_________________________ ____________________________________ ____________________________________

rs9378305 C/T *CC* *CT* *TT*  *CC* *CT* *TT*

1191 (34.15) 1682 (48.22) 615 (17.63) 0.6260 3184 (34.57) 4415 (47.94) 1611 (17.49) 0.2377

rs2741098 C/T (V69M) *CC* *CT* *TT*  *CC* *CT* *TT*

1109 (31.79) 1715 (49.17) 664 (19.04) 1.0000 2909 (31.59) 4586 (49.80) 1714 (18.61) 0.2189

rs12479626 T/C (H426R) *TT* *TC* *CC*  *TT* *TC* *CC*

3144 (90.14) 335 (9.60) 9 (0.26) 1.0000 8373 (90.91) 825 (8.96) 12 (0.13) 0.0752

rs3750208 G/A (R168W) *GG* *GA* *AA*  *GG* *GA* *AA*

2767 (79.38) 662 (18.99) 57 (1.63) 0.0206 7210 (78.34) 1871 (20.33) 122 (1.33) 0.9591

rs7809453 G/A *GG* *GA* *AA*  *GG* *GA* *AA*

1159 (33.24) 1651 (47.35) 677 (19.41) 0.0416 2995 (32.52) 4560 (49.51) 1655 (17.97) 0.2683

rs7299095 G/A *GG* *GA* *AA*  *GG* *GA* *AA*

1273 (36.51) 1636 (46.92) 578 (16.57) 0.1802 3267 (35.47) 4400 (47.78) 1543 (16.75) 0.3530

rs192236678 G/T (F521L) *GG* *GT* *TT*  *GG* *GT* *TT*

3463 (99.28) 25 (0.72) 0 (0) 1.0000 9142 (99.26) 68 (0.74) 0 (0) 1.0000

rs58098972 A/G *AA* *AG* *GG*  *AA* *AG* *GG*

2512 (72.02) 886 (25.40) 90 (2.58) 0.2661 6794 (73.77) 2206 (23.95) 210 (2.28) 0.0550

rs181990876 C/T (G450S) *CC* *CT* *TT*  *CC* *CT* *TT*

3469 (99.46) 19 (0.54) 0 (0) 1.0000 9134 (99.18) 76 (0.82) 0 (0) 1.0000

rs11171747 T/G *TT* *TG* *GG*  *TT* *TG* *GG*

1583 (45.42) 1510 (43.33) 392 (11.25) 0.2661 4166 (45.34) 3970 (43.21) 1052 (11.45) 0.0236

rs115991011 C/A (A361D) *CC* *CA* *AA*  *CC* *CA* *AA*

3448 (98.85) 40 (1.15) 0 (0) 1.0000 9119 (99.01) 91 (0.99) 0 (0) 1.0000

rs28505524 T/G *TT* *TG* *GG*  *TT* *TG* *GG*

1927 (55.26) 1335 (38.29) 225 (6.45) 0.7897 5174 (56.18) 3413 (37.06) 623 (6.76) 0.0647

rs2302445 G/A (R779H) *GG* *GA* *AA*  *GG* *GA* *AA*

1839 (52.73) 1374 (39.39) 275 (7.88) 0.4206 4854 (52.70) 3621 (39.32) 735 (7.98) 0.1008

rs807122 T/C *TT* *TC* *CC*  *TT* *TC* *CC*

2154 (61.77) 1175 (33.70) 158 (4.53) 0.9198 5746 (62.39) 3066 (33.29) 398 (4.32) 0.6828

rs138360169 T/C (N457S) *TT* *TC* *CC*  *TT* *TC* *CC*

3485 (99.91) 3 (0.09) 0 (0) 1.0000 9182 (99.70) 28 (0.30) 0 (0) 1.0000

rs2010834 A/C (F254C) *AA* *AC* *CC*  *AA* *AC* *CC*

2001 (57.37) 1272 (36.47) 215 (6.16) 0.4914 5206 (56.52) 3459 (37.56) 545 (5.92) 0.3554

rs7784072 G/C *GG* *GC* *CC*  *GG* *GC* *CC*

2990 (85.99) 462 (13.29) 25 (0.72) 0.1343 7824 (85.26) 1308 (14.25) 45 (0.49) 0.2358

rs12511068 C/T (V7I) *CC* *CT* *TT*  *CC* *CT* *TT*

2623 (75.20) 803 (23.02) 62 (1.78) 0.9414 7118 (77.30) 1951 (21.19) 139 (1.51) 0.6951

rs4731112 C/G (A357G) *CC* *CG* *GG*  *CC* *CG* *GG*

2450 (70.26) 932 (26.73) 105 (3.01) 0.1550 6534 (70.95) 2436 (26.45) 239 (2.60) 0.5060

rs145716748 A/G (S729P) *AA* *AG* *GG*  *AA* *AG* *GG*

3353 (96.13) 135 (3.87) 0 (0) 0.6406 8882 (96.44) 324 (3.52) 4 (0.04) 0.5451

rs586088 A/T (T190S) *AA* *AT* *TT*  *AA* *AT* *TT*

1630 (46.74) 1506 (43.19) 351 (10.07) 0.9066 4349 (47.22) 3913 (42.49) 948 (10.29) 0.1225

rs185067598 A/G (L277P) *AA* *AG* *GG*  *AA* *AG* *GG*

3465 (99.37) 22 (0.63) 0 (0) 1.0000 9142 (99.26) 68 (0.74) 0 (0) 1.0000

rs17150488 T/C (K381R) *TT* *TC* *CC*  *TT* *TC* *CC*

3423 (98.82) 41 (1.18) 0 (0) 1.0000 9082 (98.88) 103 (1.12) 0 (0) 1.0000

rs6762208 C/A (T301K) *CC* *CA* *AA*  *CC* *CA* *AA*

1290 (37.01) 1664 (47.73) 532 (15.26) 0.9151 3430 (37.24) 4449 (48.31) 1331 (14.45) 0.0714

rs7828656 A/C *AA* *AC* *CC*  *AA* *AC* *CC*

1025 (29.41) 1760 (50.50) 700 (20.09) 0.2742 2825 (30.68) 4617 (50.14) 1766 (19.18) 0.1231

rs6704425 C/T *CC* *CT* *TT*  *CC* *CT* *TT*

2119 (60.75) 1217 (34.89) 152 (4.36) 0.1801 5533 (60.08) 3216 (34.92) 461 (5.00) 0.8342

rs3803354 T/C *TT* *TC* *CC*  *TT* *TC* *CC*

2911 (83.46) 544 (15.60) 33 (0.94) 0.2015 7659 (83.16) 1464 (15.90) 87 (0.94) 0.0715

rs2236133 A/G *AA* *AG* *GG*  *AA* *AG* *GG*

1746 (50.07) 1420 (40.72) 321 (9.21) 0.1932 4510 (48.97) 3850 (41.80) 850 (9.23) 0.4884

rs2216317 G/A *GG* *GA* *AA*  *GG* *GA* *AA*

2254 (64.62) 1107 (31.74) 127 (3.64) 0.5536 5980 (64.93) 2846 (30.90) 384 (4.17) 0.0553

rs2302898 A/G *AA* *AG* *GG*  *AA* *AG* *GG*

2079 (59.60) 1217 (34.89) 192 (5.51) 0.4436 5310 (57.66) 3354 (36.42) 545 (5.92) 0.6085

rs7120775 C/G (Y27*) *CC* *CG* *GG*  *CC* *CG* *GG*

2450 (70.24) 932 (26.72) 106 (3.04) 0.1381 6378 (69.25) 2561 (27.81) 271 (2.94) 0.4797

rs2271862 A/G *AA* *AG* *GG*  *AA* *AG* *GG*

2324 (66.63) 1036 (29.70) 128 (3.67) 0.3406 6012 (65.28) 2845 (30.89) 353 (3.83) 0.4820

rs12807582 G/T *GG* *GT* *TT*  *GG* *GT* *TT*

941 (27.00) 1759 (50.47) 785 (22.53) 0.5189 2471 (26.84) 4650 (50.50) 2086 (22.66) 0.2596

rs6695567 A/G *AA* *AG* *GG*  *AA* *AG* *GG*

1122 (32.17) 1674 (47.99) 692 (19.84) 0.1392 2965 (32.20) 4509 (48.67) 1734 (18.83) 0.7990

rs77336780 C/G (A304G) *CC* *CG* *GG*  *CC* *CG* *GG*

2250 (64.53) 1102 (31.60) 135 (3.87) 1.0000 5816 (63.16) 2975 (32.30) 418 (4.54) 0.1356

rs141384205 G/A (R559W) *GG* *GA* *AA*  *GG* *GA* *AA*

3374 (96.73) 111 (3.18) 3 (0.09) 0.0723 8998 (97.70) 212 (2.30) 0 (0) 0.6362

rs2142662 G/A *GG* *GA* *AA*  *GG* *GA* *AA*

2616 (75.04) 814 (23.35) 56 (1.61) 0.4622 6718 (72.97) 2275 (24.71) 214 (2.32) 0.1970

rs79923436 G/A (S2302N) *GG* *GA* *AA*  *GG* *GA* *AA*

3362 (96.39) 124 (3.55) 2 (0.06) 0.3258 8864 (96.24) 336 (3.65) 10 (0.11) 0.0023

rs34685097 G/A (R236*) *GG* *GA* *AA*  *GG* *GA* *AA*

2583 (97.92) 55 (2.08) 0 (0) 1.0000 8992 (97.63) 217 (2.36) 1 (0.01) 1.0000

rs9899862 C/A (D423E) *CC* *CA* *AA*  *CC* *CA* *AA*

3067 (87.93) 412 (11.81) 9 (0.26) 0.2434 8106 (88.01) 1064 (11.55) 40 (0.44) 0.4202

rs2340917 C/T (T179M) *CC* *CT* *TT*  *CC* *CT* *TT*

998 (28.62) 1779 (51.02) 710 (20.36) 0.1091 2766 (30.04) 4481 (48.66) 1962 (21.30) 0.0646

rs1353776 G/C (E729D) *GG* *GC* *CC*  *GG* *GC* *CC*

3137 (89.94) 339 (9.72) 12 (0.34) 0.3873 8349 (90.65) 841 (9.13) 20 (0.22) 0.9090

rs34315095 C/G *CC* *CG* *GG*  *CC* *CG* *GG*

3176 (91.06) 305 (8.74) 7 (0.20) 1.0000 8340 (90.55) 846 (9.19) 24 (0.26) 0.5723

rs3748045 C/G *CC* *CG* *GG*  *CC* *CG* *GG*

1739 (49.86) 1426 (40.88) 323 (9.26) 0.2238 4331 (47.02) 3960 (43.00) 919 (9.98) 0.7535

rs1528601 C/G *CC* *CG* *GG*  *CC* *CG* *GG*

1771 (50.94) 1369 (39.37) 337 (9.69) 0.0028 4504 (48.97) 3935 (42.78) 759 (8.25) 0.0143

rs138329346 C/T (H313Y) *CC* *CT* *TT*  *CC* *CT* *TT*

3335 (95.61) 151 (4.33) 2 (0.06) 0.6889 8773 (95.26) 433 (4.70) 4 (0.04) 0.8218

rs16963698 A/G *AA* *AG* *GG*  *AA* *AG* *GG*

1017 (29.16) 1749 (50.16) 721 (20.68) 0.5619 2781 (30.20) 4545 (49.35) 1883 (20.45) 0.7523

rs200587171 C/T *CC* *CT* *TT*  *CC* *CT* *TT*

3413 (98.33) 58 (1.67) 0 (0) 1.0000 9008 (98.17) 168 (1.83) 0 (0) 1.0000

rs871443 C/T (P1779L) *CC* *CT* *TT*  *CC* *CT* *TT*

1466 (42.03) 1587 (45.50) 435 (12.47) 0.8820 3849 (41.79) 4251 (46.16) 1110 (12.05) 0.2257

rs2254067 G/T (G499C) *GG* *GT* *TT*  *GG* *GT* *TT*

2058 (59.00) 1248 (35.78) 182 (5.22) 0.7033 5613 (60.95) 3163 (34.34) 434 (4.71) 0.6919

rs140955674 G/A (T1426I) *GG* *GA* *AA*  *GG* *GA* *AA*

3078 (88.25) 402 (11.52) 8 (0.23) 0.2269 8086 (87.80) 1089 (11.82) 35 (0.38) 0.8599

rs200517965 A/T (D635V) *AA* *AT* *TT*  *AA* *AT* *TT*

3465 (99.34) 23 (0.66) 0 (0) 1.0000 9152 (99.37) 57 (0.62) 1 (0.01) 0.0890

rs78245253 G/C (A250P) *GG* *GC* *CC*  *GG* *GC* *CC*

3174 (91.00) 309 (8.86) 5 (0.14) 0.5570 8338 (90.53) 846 (9.19) 26 (0.28) 0.3671

rs10499504 A/G *AA* *AG* *GG*  *AA* *AG* *GG*

2881 (82.65) 583 (16.72) 22 (0.63) 0.2537 7623 (82.77) 1500 (16.29) 87 (0.94) 0.1654

rs10782008 G/A (V1195M) *GG* *GA* *AA*  *GG* *GA* *AA*

1587 (45.51) 1497 (42.93) 403 (11.56) 0.0849 4040 (43.87) 4105 (44.57) 1065 (11.56) 0.6584

rs117917124 C/T (V289I) *CC* *CT* *TT*  *CC* *CT* *TT*

3393 (97.28) 93 (2.66) 2 (0.06) 0.1430 8972 (97.42) 236 (2.56) 2 (0.02) 0.6699

rs10010188 C/T (A224T) *CC* *CT* *TT*  *CC* *CT* *TT*

2539 (72.83) 863 (24.76) 84 (2.41) 0.3130 6652 (72.30) 2307 (25.08) 241 (2.62) 0.0186

rs2588941 C/T *CC* *CT* *TT*  *CC* *CT* *TT*

925 (26.52) 1771 (50.77) 792 (22.71) 0.3255 2571 (27.91) 4544 (49.34) 2095 (22.75) 0.3158

rs10794531 C/T (R53H) *CC* *CT* *TT*  *CC* *CT* *TT*

1363 (39.08) 1589 (45.55) 536 (15.37) 0.0409 3635 (39.47) 4277 (46.44) 1298 (14.09) 0.4899

rs12675375 C/T (G337D) *CC* *CT* *TT*  *CC* *CT* *TT*

1114 (32.19) 1662 (48.02) 685 (19.79) 0.1472 2998 (32.65) 4466 (48.65) 1717 (18.70) 0.4563

rs265654 A/G *AA* *AG* *GG*  *AA* *AG* *GG*

1021 (29.30) 1729 (49.61) 735 (21.09) 0.9456 2638 (28.64) 4609 (50.04) 1963 (21.32) 0.5574

rs1959607 T/C *TT* *TC* *CC*  *TT* *TC* *CC*

3383 (96.99) 103 (2.95) 2 (0.06) 0.1948 8931 (96.99) 274 (2.98) 3 (0.03) 0.4726

rs6923504 C/G *CC* *CG* *GG*  *CC* *CG* *GG*

2022 (57.99) 1246 (35.73) 219 (6.28) 0.1518 5446 (59.13) 3232 (35.09) 532 (5.78) 0.0709

rs35928055 A/G (S240G) *AA* *AG* *GG*  *AA* *AG* *GG*

2675 (76.69) 750 (21.50) 63 (1.81) 0.2172 7117 (77.28) 1938 (21.04) 155 (1.68) 0.0886

rs113710653 C/T (E231K) *CC* *CT* *TT*  *CC* *CT* *TT*

1449 (93.91) 94 (6.09) 0 (0) 0.3980 6918 (97.69) 161 (2.27) 3 (0.04) 0.0742

rs13093808 C/A (A561E) *CC* *CA* *AA*  *CC* *CA* *AA*

2488 (71.33) 914 (26.20) 86 (2.47) 0.8468 6527 (70.87) 2453 (26.63) 230 (2.50) 1.0000

rs200121865 G/C (G149A) *GG* *GC* *CC*  *GG* *GC* *CC*

3454 (99.02) 33 (0.95) 1 (0.03) 0.0821 9165 (99.51) 45 (0.49) 0 (0) 1.0000

rs2303146 A/C (C91W) *AA* *AC* *CC*  *AA* *AC* *CC*

1019 (29.22) 1734 (49.71) 735 (21.07) 0.9728 2763 (30.00) 4548 (49.38) 1899 (20.62) 0.7366

rs3828923 G/A *GG* *GA* *AA*  *GG* *GA* *AA*

3088 (88.56) 382 (10.95) 17 (0.49) 0.1720 8010 (86.98) 1155 (12.54) 44 (0.48) 0.7402

rs1233397 C/T *CC* *CT* *TT*  *CC* *CT* *TT*

826 (26.55) 1720 (49.31) 842 (24.14) 0.4359 2552 (27.71) 4546 (49.36) 2112 (22.93) 0.3160

rs74491133 C/T *CC* *CT* *TT*  *CC* *CT* *TT*

3385 (97.05) 103 (2.95) 0 (0) 1.0000 8964 (97.34) 243 (2.64) 2 (0.02) 0.6816

rs6738031 A/C (I958M) *AA* *AC* *CC*  *AA* *AC* *CC*

1721 (49.34) 1439 (41.26) 328 (9.40) 0.2769 4587 (49.81) 3790 (41.15) 833 (9.04) 0.2115

rs3748993 C/A (P586T) *CC* *CA* *AA*  *CC* *CA* *AA*

2231 (64.05) 1102 (31.64) 150 (4.31) 0.3430 6018 (65.51) 2817 (30.66) 352 (3.83) 0.3287

rs12654264 T/A *TT* *TA* *AA*  *TT* *TA* *AA*

920 (26.38) 1723 (49.40) 845 (24.22) 0.4981 2454 (26.65) 4608 (50.03) 2148 (23.32) 0.8839

rs36088178 T/C (N79S) *TT* *TC* *CC*  *TT* *TC* *CC*

3123 (89.54) 357 (10.23) 8 (0.23) 0.6169 8310 (90.23) 882 (9.58) 18 (0.19) 0.3224

rs2395253 G/A *GG* *GA* *AA*  *GG* *GA* *AA*

3354 (96.27) 126 (3.62) 4 (0.11) 0.0378 8870 (96.64) 299 (3.26) 9 (0.10) 0.0016

rs11466651 C/T (V298I) *CC* *CT* *TT*  *CC* *CT* *TT*

2807 (80.48) 633 (18.15) 48 (1.37) 0.0848 7452 (80.91) 1642 (17.83) 116 (1.26) 0.0224

rs2501279 C/T *CC* *CT* *TT*  *CC* *CT* *TT*

1789 (51.29) 1414 (40.54) 285 (8.17) 0.8030 4496 (48.82) 3923 (42.59) 791 (8.59) 0.1231

rs202069030 G/C (R51S) *GG* *GC* *CC*  *GG* *GC* *CC*

3449 (99.88) 4 (0.12) 0 (0) 1.0000 9039 (98.83) 107 (1.17) 0 (0) 1.0000

rs2271251 C/G (A82G) *CC* *CG* *GG*  *CC* *CG* *GG*

3416 (97.94) 71 (2.03) 1 (0.03) 0.3166 9089 (98.70) 119 (1.29) 1 (0.01) 0.3275

rs663824 A/G (N241D) *AA* *AG* *GG*  *AA* *AG* *GG*

2046 (58.66) 1251 (35.87) 191 (5.47) 1.0000 5355 (58.14) 3321 (36.06) 534 (5.80) 0.5277

rs147317864 C/T (A262T) *CC* *CT* *TT*  *CC* *CT* *TT*

2536 (99.41) 15 (0.59) 0 (0) 1.000 7678 (100.00) 0 (0) 0 (0) ND

rs7188 T/G *TT* *TG* *GG*  *TT* *TG* *GG*

1634 (46.86) 1551 (44.48) 302 (8.66) 0.0155 4245 (46.09) 3949 (42.88) 1016 (11.03) 0.0325

rs201522765 C/T (P151L) *CC* *CT* *TT*  *CC* *CT* *TT*

3462 (99.26) 26 (0.74) 0 (0) 1.0000 9148 (99.34) 61 (0.66) 0 (0) 1.0000

rs1057251 T/C (V867A) *TT* *TC* *CC*  *TT* *TC* *CC*

3279 (94.01) 204 (5.85) 5 (0.14) 0.3812 8625 (93.65) 571 (6.20) 14 (0.15) 0.1807

rs2838497 C/G (L217V) *CC* *CG* *GG*  *CC* *CG* *GG*

2602 (74.60) 815 (23.37) 71 (2.03) 0.4317 6907 (75.00) 2121 (23.03) 181 (1.97) 0.2276

rs6773957 A/G *AA* *AG* *GG*  *AA* *AG* *GG*

1205 (34.56) 1605 (46.03) 677 (19.41) 0.0007 2966 (32.20) 4485 (48.70) 1759 (19.10) 0.3847

rs12185961 G/A *GG* *GA* *AA*  *GG* *GA* *AA*

1962 (56.25) 1315 (37.70) 211 (6.05) 0.6510 5283 (57.36) 3403 (36.95) 524 (5.69) 0.4428

rs8409 G/A *GG* *GA* *AA*  *GG* *GA* *AA*

997 (28.58) 1762 (50.52) 729 (20.90) 0.3404 2685 (29.15) 4611 (50.07) 1914 (20.78) 0.4252

rs80006813 A/C (K589Q) *AA* *AC* *CC*  *AA* *AC* *CC*

3481 (99.80) 7 (0.20) 0 (0) 1.0000 9170 (99.58) 39 (0.42) 0 (0) 1.0000

rs3740955 G/A (H249R) *GG* *GA* *AA*  *GG* *GA* *AA*

1997 (57.27) 1281 (36.74) 209 (5.99) 0.8542 5429 (58.95) 3273 (35.54) 507 (5.51) 0.6406

rs3210400 G/A (A183T) *GG* *GA* *AA*  *GG* *GA* *AA*

1903 (54.56) 1328 (38.07) 257 (7.67) 0.2394 4900 (53.20) 3578 (38.85) 732 (7.95) 0.0296

rs1611196 T/C *TT* *TC* *CC*  *TT* *TC* *CC*

1817 (60.34) 1064 (35.34) 130 (4.32) 0.1108 5913 (66.27) 2701 (30.27) 309 (3.46) 0.9721

rs74887188 T/C (I282V) *TT* *TC* *CC*  *TT* *TC* *CC*

2790 (79.99) 659 (18.89) 39 (1.12) 1.0000 7247 (78.69) 1838 (19.96) 124 (1.35) 0.5336

rs2271395 A/G (T1587A) *AA* *AG* *GG*  *AA* *AG* *GG*

1083 (31.22) 1697 (48.92) 689 (19.86) 0.6059 2626 (28.58) 4561 (49.64) 2001 (21.78) 0.8176

rs143803280 G/A *GG* *GA* *AA*  *GG* *GA* *AA*

3468 (99.43) 19 (0.54) 1 (0.03) 0.0297 9175 (99.62) 35 (0.38) 0 (0) 1.0000

rs61729488 T/C (N771S) *TT* *TC* *CC*  *TT* *TC* *CC*

3370 (96.62) 118 (3.38) 0 (0) 0.6258 8889 (96.53) 315 (3.42) 5 (0.05) 0.2128

rs36101975 C/T *CC* *CT* *TT*  *CC* *CT* *TT*

2741 (78.58) 698 (20.01) 49 (1.41) 0.5573 7206 (78.24) 1895 (20.58) 109 (1.18) 0.2377

rs75289680 T/G (V45G) *TT* *TG* *GG*  *TT* *TG* *GG*

3389 (97.19) 98 (2.81) 0 (0) 1.0000 8916 (96.81) 292 (3.17) 2 (0.02) 1.0000

rs33972313 C/T (V264M) *CC* *CT* *TT*  *CC* *CT* *TT*

3467 (99.40) 20 (0.57) 1 (0.03) 0.0327 9129 (99.12) 80 (0.87) 1 (0.01) 0.1656

rs78198420 A/T (N271Y) *AA* *AT* *TT*  *AA* *AT* *TT*

3404 (97.59) 83 (2.38) 1 (0.03) 0.4043 8937 (97.08) 266 (2.89) 3 (0.03) 0.4549

rs3739451 A/T (I3161F) *AA* *AT* *TT*  *AA* *AT* *TT*

2912 (83.49) 552 (15.82) 24 (0.69) 0.8291 7689 (83.48) 1455 (15.80) 66 (0.72) 0.8425

rs148815814 C/T (R592Q) *CC* *CT* *TT*  *CC* *CT* *TT*

3472 (99.54) 16 (0.46) 0 (0) 1.0000 9168 (99.54) 42 (0.46) 0 (0) 1.0000

rs72553867 C/A (T94K) *CC* *CA* *AA*  *CC* *CA* *AA*

2444 (70.21) 959 (27.55) 78 (2.24) 0.1658 6593 (71.64) 2378 (25.84) 232 (2.52) 0.3182

rs192670611 C/T (R915C) *CC* *CT* *TT*  *CC* *CT* *TT*

3466 (99.37) 22 (0.63) 0 (0) 1.0000 9169 (99.56) 41 (0.44) 0 (0) 1.0000

rs308341 G/A *GG* *GA* *AA*  *GG* *GA* *AA*

2182 (62.56) 1151 (33.00) 155 (4.44) 0.8380 5695 (61.84) 3107 (33.73) 408 (4.43) 0.5549

rs806276 A/G *AA* *AG* *GG*  *AA* *AG* *GG*

2240 (64.27) 1081 (31.02) 164 (4.71) 0.0240 5899 (64.05) 2933 (31.90) 373 (4.05) 0.7695

rs10100485 G/A *GG* *GA* *AA*  *GG* *GA* *AA*

1305 (37.41) 1636 (46.91) 547 (15.68) 0.3742 3700 (40.17) 4232 (45.95) 1278 (13.88) 0.2267

rs201752702 T/C (D133G) *TT* *TC* *CC*  *TT* *TC* *CC*

3481 (99.80) 7 (0.20) 0 (0) 1.0000 9193 (99.84) 15 (0.16) 0 (0) 1.0000

rs28516377 C/T (G826S) *CC* *CT* *TT*  *CC* *CT* *TT*

1685 (48.31) 1470 (42.14) 333 (9.55) 0.6326 4467 (48.50) 3914 (42.50) 829 (9.00) 0.5050

rs201586390 C/T (R458H) *CC* *CT* *TT*  *CC* *CT* *TT*

3415 (97.91) 73 (2.09) 0 (0) 1.0000 9021 (97.95) 188 (2.04) 1 (0.01) 0.6265

rs799889 C/A *CC* *CA* *AA*  *CC* *CA* *AA*

1216 (34.86) 1660 (47.59) 612 (17.55) 0.2791 3107 (33.73) 4441 (48.22) 1662 (18.05) 0.2854

rs118079207 G/A (R716H) *GG* *GA* *AA*  *GG* *GA* *AA*

3427 (98.25) 61 (1.75) 0 (0) 1.0000 9082 (98.61) 128 (1.39) 0 (0) 1.0000

rs2251220 G/A (S849L) *GG* *GA* *AA*  *GG* *GA* *AA*

2381 (68.26) 994 (28.50) 113 (3.24) 0.4460 6219 (67.52) 2702 (29.34) 289 (3.14) 0.8587

rs362726 T/C *TT* *TC* *CC*  *TT* *TC* *CC*

1006 (28.85) 1724 (49.44) 757 (21.71) 0.7335 2708 (29.40) 4528 (49.17) 1974 (21.43) 0.3142

rs11962089 A/G *AA* *AG* *GG*  *AA* *AG* *GG*

3154 (90.43) 328 (9.40) 6 (0.17) 0.5809 8376 (90.94) 815 (8.85) 19 (0.21) 1.0000

rs201776800 T/C (M465T) *TT* *TC* *CC*  *TT* *TC* *CC*

3154 (90.42) 325 (9.32) 9 (0.26) 0.8552 8394 (91.14) 799 (8.68) 17 (0.18) 0.8087

rs117463303 G/A *GG* *GA* *AA*  *GG* *GA* *AA*

3373 (96.73) 113 (3.24) 1 (0.03) 0.6154 8865 (96.26) 343 (3.72) 2 (0.02) 0.7745

rs801524 A/G *AA* *AG* *GG*  *AA* *AG* *GG*

1272 (36.49) 1645 (47.19) 569 (16.32) 0.3405 3222 (34.99) 4424 (48.04) 1563 (16.97) 0.5043

rs714106 A/C (T121N) *AA* *AC* *CC*  *AA* *AC* *CC*

1030 (29.55) 1684 (48.31) 772 (22.14) 0.0951 2722 (29.55) 4531 (49.20) 1957 (21.25) 0.3781

rs3732813 T/C (T406A) *TT* *TC* *CC*  *TT* *TC* *CC*

2058 (59.00) 1232 (35.32) 198 (5.68) 0.4490 5317 (57.73) 3324 (36.09) 569 (6.18) 0.1057

rs57006145 T/G (T824P) *TT* *TG* *GG*  *TT* *TG* *GG*

2963 (84.95) 503 (14.42) 22 (0.63) 0.9065 7959 (86.42) 1198 (13.01) 53 (0.57) 0.2677

rs141567247 C/T (G295R) *CC* *CT* *TT*  *CC* *CT* *TT*

3395 (97.33) 90 (2.58) 3 (0.09) 0.0267 9011 (97.84) 199 (2.16) 0 (0) 0.6293

rs151285112 T/C (T38A) *TT* *TC* *CC*  *TT* *TC* *CC*

3362 (96.39) 123 (3.53) 3 (0.08) 0.1137 8817 (95.73) 390 (4.24) 3 (0.03) 0.8021

rs45554335 A/C (D461A) *AA* *AC* *CC*  *AA* *AC* *CC*

1258 (36.07) 1656 (47.48) 574 (16.45) 0.4597 3507 (38.08) 4374 (47.49) 1329 (14.43) 0.5661

rs2076671 T/C (M272T) *TT* *TC* *CC*  *TT* *TC* *CC*

1076 (30.85) 1748 (50.11) 664 (19.04) 0.3364 2827 (30.70) 4638 (50.36) 1744 (18.94) 0.0425

____________________________________________________________________________________________________________

Data are numbers of subjects (percentages). H-W *P*, *P* value for Hardy-Weinberg equilibrium. ND, not determined.

**Supplementary Table 3.** Relation of single nucleotide polymorphisms (SNPs) to coronary artery disease as determined by multivariable logistic regression analysis.

__________________________________________________________________________________________________________________________

SNP Dominant Recessive Additive 1 Additive 2

____________________ ____________________ ____________________ ____________________

*P* OR (95% CI) *P* OR (95% CI) *P* OR (95% CI) *P* OR (95% CI)

__________________________________________________________________________________________________________________________

rs9378305 C/T 0.1423 0.5749 0.0791 0.7817

rs2741098 C/T (V69M) 0.4085 0.5192 0.2715 0.9611

rs12479626 T/C (H426R) 0.2019 0.4729 0.2310 0.4639

rs3750208 G/A (R168W) 0.1733 0.3120 0.0977 0.3637

rs7809453 G/A 0.1997 0.2027 0.0710 0.7698

rs7299095 G/A 0.8567 0.2370 0.8241 0.3302

rs192236678 G/T (F521L) 0.9870 ND 0.9870 ND

rs58098972 A/G 0.0064 1.16 (1.04–1.29) 0.2601 0.0118 1.15 (1.03–1.29) 0.1780

rs181990876 C/T (G450S) 0.2650 ND 0.2650 ND

rs11171747 T/G 0.7840 0.9100 0.8018 0.8527

rs115991011 C/A (A361D) 0.4533 ND 0.4533 ND

rs28505524 T/G 0.6493 0.8481 0.6769 0.7845

rs2302445 G/A (R779H) 0.6106 0.7011 0.5079 0.8285

rs807122 T/C 0.6633 0.6849 0.7376 0.6506

rs138360169 T/C (N457S) 0.0460 0.32 (0.07–0.98) ND 0.0460 0.32 (0.07–0.98) ND

rs2010834 A/C (F254C) 0.4647 0.1591 0.2328 0.2495

rs7784072 G/C 0.3411 0.0223 2.01 (1.11–3.55) 0.1575 0.0251 1.98 (1.09–3.50)

rs12511068 C/T (V7I) 0.1464 0.1767 0.2396 0.1532

rs4731112 C/G (A357G) 0.3203 0.5817 0.3791 0.5237

rs145716748 A/G (S729P) 0.9308 0.0696 0.8111 0.0697

rs586088 A/T (T190S) 0.4182 0.9332 0.3779 0.8584

rs185067598 A/G (L277P) 0.3033 ND 0.3033 ND

rs17150488 T/C (K381R) 0.7181 ND 0.7181 ND

rs6762208 C/A (T301K) 0.2925 0.3845 0.4133 0.2611

rs7828656 A/C 0.0190 1.13 (1.02–1.26) 0.1896 0.0424 1.12 (1.00–1.25) 0.0320 1.16 (1.01–1.34)

rs6704425 C/T 0.4299 0.8692 0.4390 0.7727

rs3803354 T/C 0.7528 0.8612 0.7772 0.8515

rs2236133 A/G 0.1595 0.9633 0.1428 0.6645

rs2216317 G/A 0.6783 0.5490 0.5464 0.6086

rs2302898 A/G 0.0321 0.90 (0.82–0.99) 0.6606 0.0350 0.90 (0.81–0.99) 0.4081

rs7120775 C/G (Y27*) 0.5564 0.5698 0.6509 0.5392

rs2271862 A/G 0.2446 0.7978 0.2557 0.6873

rs12807582 G/T 0.7189 0.7292 0.6162 0.9862

rs6695567 A/G 0.9438 0.2726 0.7510 0.4130

rs77336780 C/G (A304G) 0.0355 0.90 (0.81–0.99) 0.0145 0.74 (0.57–0.94) 0.1381 0.0085 0.72 (0.55–0.92)

rs141384205 G/A (R559W) 0.2079 0.0526 0.2858 0.0521

rs2142662 G/A 0.0323 0.89 (0.79–0.99) 0.0289 0.67 (0.46–0.96) 0.0943 0.0208 0.66 (0.45–0.94)

rs79923436 G/A (S2302N) 0.5514 0.4688 0.6325 0.4669

rs34685097 G/A (R236*) 0.3463 0.3217 0.3783 0.3211

rs9899862 C/A (D423E) 0.8175 0.6930 0.8675 0.6903

rs2340917 C/T (T179M) 0.0281 1.13 (1.01–1.25) 0.8374 0.0164 1.15 (1.03–1.28) 0.2943

rs1353776 G/C (E729D) 0.2971 0.6289 0.3294 0.6168

rs34315095 C/G 0.9921 0.4902 0.9033 0.4915

rs3748045 C/G 0.0778 0.9675 0.0651 0.5812

rs1528601 C/G 0.5442 0.0104 1.25 (1.05–1.47) 0.1413 0.0370 1.20 (1.01–1.43)

rs138329346 C/T (H313Y) 0.7095 0.9891 0.7091 0.9877

rs16963698 A/G 0.8396 0.5825 0.6835 0.7907

rs200587171 C/T 0.6824 ND 0.6824 ND

rs871443 C/T (P1779L) 0.3597 0.6168 0.4244 0.4576

rs2254067 G/T (G499C) 0.0597 0.2219 0.1082 0.1406

rs140955674 G/A (T1426I) 0.5149 0.1243 0.6700 0.1222

rs200517965 A/T (D635V) 0.4959 0.5580 0.5255 0.5579

rs78245253 G/C (A250P) 0.5085 0.3807 0.5876 0.3765

rs10499504 A/G 0.3742 0.5769 0.3089 0.6054

rs10782008 G/A (V1195M) 0.1603 0.7030 0.1758 0.4273

rs117917124 C/T (V289I) 0.3506 0.9529 0.3446 0.9552

rs10010188 C/T (A224T) 0.7635 0.8980 0.7287 0.9245

rs2588941 C/T 0.4675 0.9770 0.4345 0.6919

rs10794531 C/T (R53H) 0.9493 0.2516 0.7389 0.3511

rs12675375 C/T (G337D) 0.8643 0.9093 0.8882 0.8675

rs265654 A/G 0.4756 0.7233 0.5278 0.5281

rs1959607 T/C 0.9512 0.2919 0.8419 0.2923

rs6923504 C/G 0.5852 0.3158 0.7971 0.3014

rs35928055 A/G (S240G) 0.5953 0.5666 0.4896 0.5998

rs113710653 C/T (E231K) 0.4142 0.4863 0.4413 0.4855

rs13093808 C/A (A561E) 0.3234 0.9757 0.3045 0.9465

rs200121865 G/C (G149A) 0.0034 2.27 (1.32–3.86) 0.0682 0.0065 2.16 (1.25–3.69) 0.0679

rs2303146 A/C (C91W) 0.9125 0.7918 0.9812 0.8108

rs3828923 G/A 0.1407 0.5959 0.1096 0.6244

rs1233397 C/T 0.4028 0.4207 0.5484 0.3159

rs74491133 C/T 0.6938 0.5645 0.6761 0.5648

rs6738031 A/C (I958M) 0.4796 0.8815 0.4278 0.9450

rs3748993 C/A (P586T) 0.2667 0.9746 0.2522 0.8507

rs12654264 T/A 0.4611 0.7638 0.4995 0.5334

rs36088178 T/C (N79S) 0.7588 0.5257 0.6938 0.5294

rs2395253 G/A 0.6886 0.0396 0.15 (0.01–0.92) 0.9448 0.0396 0.15 (0.01–0.92)

rs11466651 C/T (V298I) 0.8859 0.4677 0.9801 0.4691

rs2501279 C/T 0.2004 0.9720 0.1829 0.7025

rs202069030 G/C (R51S) 0.0099 0.32 (0.09–0.78) ND 0.0099 0.32 (0.09–0.78) ND

rs2271251 C/G (A82G) 0.0304 1.49 (1.04–2.13) 0.4837 0.0363 1.48 (1.03–2.11) 0.4819

rs663824 A/G (N241D) 0.6617 0.4527 0.8176 0.4351

rs147317864 C/T (A262T) 0.0255 >100 (ND) ND 0.0255 >100 (ND) ND

rs7188 T/G 0.5784 0.0009 0.76 (0.64–0.89) 0.6171 0.0025 0.77 (0.65–0.91)

rs201522765 C/T (P151L) 0.4127 ND 0.4127 ND

rs1057251 T/C (V867A) 0.7534 0.8171 0.7804 0.8147

rs2838497 C/G (L217V) 0.2408 0.7380 0.2603 0.6741

rs6773957 A/G 0.1159 0.7712 0.0743 0.5689

rs12185961 G/A 0.7159 0.4942 0.8607 0.4810

rs8409 G/A 0.4874 0.7965 0.5185 0.5809

rs80006813 A/C (K589Q) 0.0328 0.37 (0.12–0.93) ND 0.0328 0.37 (0.12–0.93) ND

rs3740955 G/A (H249R) 0.2046 0.4721 0.2684 0.3620

rs3210400 G/A (A183T) 0.3883 0.4555 0.5076 0.3802

rs1611196 T/C 0.0736 0.2262 0.1284 0.1655

rs74887188 T/C (I282V) 0.1026 0.3794 0.1408 0.3367

rs2271395 A/G (T1587A) 0.0023 0.85 (0.77–0.94) 0.0918 0.0084 0.86 (0.77–0.96) 0.0052 0.82 (0.72–0.94)

rs143803280 G/A 0.0615 ND 0.0615 ND

rs61729488 T/C (N771S) 0.3706 0.1567 0.4313 0.1561

rs36101975 C/T 0.8625 0.6963 0.7884 0.7087

rs75289680 T/G (V45G) 0.1526 0.3651 0.1668 0.3638

rs33972313 C/T (V264M) 0.2432 0.9186 0.2268 0.9199

rs78198420 A/T (N271Y) 0.0487 0.74 (0.54–1.00) 0.3994 0.0539 0.3977

rs3739451 A/T (I3161F) 0.8309 0.6286 0.9046 0.6255

rs148815814 C/T (R592Q) 0.9239 ND 0.9239 ND

rs72553867 C/A (T94K) 0.2806 0.9608 0.2600 0.9537

rs192670611 C/T (R915C) 0.2009 ND 0.2009 ND

rs308341 G/A 0.4444 0.7675 0.3795 0.8739

rs806276 A/G 0.4760 0.2824 0.2885 0.3637

rs10100485 G/A 0.0117 1.14 (1.03–1.25) 0.0510 0.0471 1.11 (1.00–1.23) 0.0104 1.21 (1.05–1.40)

rs201752702 T/C (D133G) 0.9660 ND 0.9660 ND

rs28516377 C/T (G826S) 0.5393 0.9681 0.5106 0.8899

rs201586390 C/T (R458H) 0.7529 0.4210 0.7173 0.4212

rs799889 C/A 0.3781 0.9567 0.3596 0.6477

rs118079207 G/A (R716H) 0.4253 ND 0.4253 ND

rs2251220 G/A (S849L) 0.3669 0.5198 0.2655 0.6107

rs362726 T/C 0.9100 0.6191 0.7662 0.7844

rs11962089 A/G 0.0694 0.4359 0.0520 0.4522

rs201776800 T/C (M465T) 0.2519 0.1552 0.3395 0.1514

rs117463303 G/A 0.3583 0.6722 0.3362 0.6750

rs801524 A/G 0.7133 0.5028 0.8772 0.5016

rs714106 A/C (T121N) 0.9953 0.3960 0.7543 0.5710

rs3732813 T/C (T406A) 0.0880 0.2848 0.1459 0.1854

rs57006145 T/G (T824P) 0.4349 0.5158 0.3562 0.5348

rs141567247 C/T (G295R) 0.0181 1.45 (1.07–1.96) 0.0233 >100 (ND) 0.0342 1.41 (1.03–1.91) 0.0230 >100 (ND)

rs151285112 T/C (T38A) 0.1090 0.3502 0.0823 0.3554

rs45554335 A/C (D461A) 0.1089 0.0747 0.2795 0.0387 1.17 (1.01–1.35)

rs2076671 T/C (M272T) 0.7543 0.7709 0.8170 0.7134

__________________________________________________________________________________________________________________________

Multivariable logistic regression analysis was performed with adjustment for age, sex, and the prevalence of hypertension, diabetes mellitus, and dyslipidemia. Based on Bonferroni’s correction, a *P* value of <9.92 × 10^–5^ (0.05/504) was considered statistically significant. OR, odds ratio; CI, confidence interval; ND, not determined.

**Supplementary Table 4.** The 114 single nucleotide polymorphisms (SNPs) significantly (*P* < 1.21 × 10^–6^) associated with myocardial infarction in the exome-wide association study.

___________________________________________________________________________________

Gene dbSNP Nucleotide Chromosome: MAF *P* (allele) Allele

(amino acid) position (%) OR

substitution^a^

___________________________________________________________________________________

*THAP11* rs75778351 C/G (L157V) 16: 67843023 0.1 6.56 × 10^–218^ 0.69

*TRIM7* rs116911833 G/A (T80M) 5: 181199104 2.0 4.74 × 10^–213^ 0.86

*MYO7B* rs4662742 C/A 2: 127593169 28.5 1.88 × 10^–209^ 1.00

*FAM26F* rs41289932 C/G (N310K) 6: 116463687 4.6 1.11 × 10^–186^ 1.07

*DDIT4L* rs11726613 A/C 4: 100186900 37.4 2.65 × 10^–160^ 0.95

*ABTB1* rs117417589 A/G (K406E) 3: 127680171 2.0 1.39 × 10^–158^ 1.18

*CWH43* rs202103723 C/A (P511Q) 4: 49032589 0.3 2.38 × 10^–147^ 2.63

*C5* rs56188613 C/A (G451V) 9: 121020130 0.9 9.03 × 10^–144^ 0.93

rs6557316 G/A 6: 153772131 48.0 7.85 × 10^–139^ 0.98

rs3135365 T/G 6: 32421478 18.9 7.74 × 10^–111^ 0.95

*UTP2* rs193164904 A/G (I534V) 16: 69163131 0.2 5.92 × 10^–99^ 0.88

rs11624336 G/A 14: 96727175 14.9 4.02 × 10^–94^ 0.95

*NDUFAF1* rs3204853 C/A (R31L) 15: 41396968 1.4 4.65 × 10^–82^ 1.08

*KDM1B* rs41267732 A/T (M416L) 6: 18212563 0.9 9.60 × 10^–82^ 0.88

*CMIP* rs2925979 G/A 16: 81501185 33.7 1.05 × 10^–81^ 1.01

*DNAH11* rs6965750 G/A 7: 21784504 4.4 4.47 × 10^–79^ 0.98

*HMGCS2* rs181428774 G/T (L59M) 1: 119764556 0.3 4.42 × 10^–78^ 0.85

*FAT2* rs9324700 A/G (F686S) 5: 151566875 29.2 1.16 × 10^–74^ 0.92

*SLC22A3* rs7758229 G/T 6: 160419220 24.1 8.17 × 10^–72^ 1.01

*TCEB3B* rs2010834 A/C (F254C) 18: 47034504 24.5 1.92 × 10^–68^ 0.97

*ALDH3B2* rs1551888 T/C (N52S) 11: 67666398 5.0 1.32 × 10^–61^ 0.93

*PCNX2* rs56231757 C/T (S1901N) 1: 232998340 10.5 2.01 × 10^–61^ 1.08

*SMCR8* rs1563631 C/T 17: 18317820 8.4 2.28 × 10^–61^ 0.95

*MMP28* rs79742527 G/A 17: 35766662 13.6 4.33 × 10^–60^ 1.03

*MAPKBP1* rs148608964 C/T (P1241L) 15: 41823552 1.6 3.90 × 10^–58^ 0.89

*STXBP2* rs188212047 G/T (L212F) 19: 7642058 0.8 7.43 × 10^–57^ 2.51

*VPS13D* rs143833298 G/A (R830Q) 1: 12276077 0.8 1.90 × 10^–54^ 0.76

*KIAA1324* rs1052878 C/T (P922L) 1: 109202996 4.9 9.97 × 10^–52^ 0.83

*AXDND1* rs201169529 A/G (Y790C) 1: 179492932 0.6 1.01 × 10^–49^ 1.13

*CCHCR1* rs1265110 G/A 6: 31151645 30.2 1.30 × 10^–48^ 0.87

*OR51I1* rs77336780 C/G (A304G) 11: 5440604 20.6 1.16 × 10^–47^ 0.92

rs1480347 G/A 8: 20489946 17.3 1.94 × 10^–47^ 0.93

*ABAT* rs11641035 G/A 16: 8763892 14.5 1.71 × 10^–44^ 1.02

*NCAPG2* rs80143472 G/C (E952D) 7: 158652371 7.3 9.19 × 10^–42^ 1.03

*ALDH16A1* rs2293012 T/G (M9L) 19: 49451550 30.5 4.51 × 10^–41^ 1.12

*CLCA4* rs2231599 A/G (S492G) 1: 86574546 1.1 1.95 × 10^–40^ 1.11

*NEK5* rs12146904 G/A 13: 52126334 44.7 1.40 × 10^–36^ 0.99

*UBXN11* rs138559558 G/A (R289C) 1: 26284470 1.2 3.07 × 10^–32^ 1.08

*MKRN2* rs2305296 A/G (I36V) 3: 12568954 2.1 7.60 × 10^–32^ 1.06

*ABCB8* rs56198402 G/A (V454I) 7: 151041967 0.7 9.95 × 10^–32^ 1.03

*S100A3* rs36022742 C/T (R3K) 1: 153548478 1.6 9.95 × 10^–32^ 1.03

*SCMH1* rs182666831 A/T (S48C) 1: 41151649 0.1 2.22 × 10^–30^ 0.76

*ASB13* rs138695721 A/C (V139G) 10: 5649071 0.6 3.79 × 10^–29^ 0.65

*OR10G2* rs41314525 C/A (R85L) 14: 21634589 39.5 7.63 × 10^–29^ 1.00

rs11007350 C/T 10: 29000102 23.8 4.68 × 10^–28^ 0.90

rs7752978 A/G 6: 114869897 47.4 4.06 × 10^–27^ 1.00

rs1910795 C/T 5: 26809806 49.8 9.25 × 10^–25^ 0.98

*TP63* rs10937405 C/T 3: 189665394 31.8 1.82 × 10^–24^ 0.96

*PRICKLE1* rs34837068 A/G (V125A) 12: 42469460 4.9 3.36 × 10^–20^ 0.95

*FCRLA* rs117378923 G/A (V144M) 1: 161712080 0.6 1.67 × 10^–19^ 0.80

*PIK3C2B* rs17847749 G/C (W691C) 1: 204450011 0.4 2.66 × 10^–19^ 1.34

*LRRC17* rs1057066 G/C (G187A) 7: 102934473 29.6 1.45 × 10^–17^ 1.02

*ABCB4* rs45476795 G/T (T651N) 7: 87426862 0.8 1.49 × 10^–15^ 0.90

*CFAP61* rs114335521 A/G (Y203C) 20: 20090885 0.5 1.14 × 10^–13^ 0.99

*LIPT2* rs586088 A/T (T190S) 11: 74492263 31.5 1.23 × 10^–13^ 1.00

rs13223723 T/C 7: 99955269 12.9 1.33 × 10^–13^ 1.05

*AP1G1* rs904763 G/T (P685H) 16: 71739287 32.0 2.19 × 10^–12^ 0.98

*ABCA2* rs12348881 T/G 9: 137017531 3.4 2.28 × 10^–12^ 1.04

*CORO1C* rs201986662 T/C (I472M) 12: 108647412 0.3 2.64 × 10^–12^ 2.00

*ZNF683* rs10794531 C/T (R53H) 1: 26367754 37.7 4.49 × 10^–12^ 1.05

*ITPK1* rs143953605 C/T (V55I) 14: 93016759 0.9 4.72 × 10^–12^ 0.83

*CASC17* rs1859962 T/G 17: 71112612 25.7 4.75 × 10^–12^ 0.97

*KRT2* rs2634041 C/T (G101S) 12: 52651842 24.7 5.67 × 10^–12^ 1.04

*DNAH8* rs200536426 G/A (D4519N) 6: 39012478 0.5 1.38 × 10^–11^ 0.65

*IQGAP2* rs9293688 T/C 5: 76543340 34.2 1.98 × 10^–11^ 1.00

*USH2A* rs192115090 G/C (A2210P) 1: 215998916 0.8 1.99 × 10^–11^ 1.20

rs1233397 C/T 6: 29577938 47.6 2.07 × 10^–11^ 1.05

rs2745400 G/A 6: 29520472 46.3 2.13 × 10^–11^ 0.99

rs9258102 T/C 6: 29701136 10.8 4.84 × 10^–11^ 1.20

rs2643184 A/G 3: 164686504 41.2 9.76 × 10^–11^ 0.97

*SPC24* rs74491133 C/T 19: 11147894 1.3 1.20 × 10^–10^ 1.09

*OR9G1* rs79060400 T/C 11: 56701218 13.7 1.70 × 10^–10^ 1.07

*ROR2* rs200867550 C/T (V874I) 9: 91723874 0.2 2.11 × 10^–10^ 0.22

*OLFML2A* rs16927649 G/A (R425Q) 9: 124807886 1.3 5.12 × 10^–10^ 1.10

*EMILIN2* rs56288451 C/T (P903S) 18: 2909702 14.5 8.83 × 10^–10^ 1.03

*ACOT11* rs1368883 G/A (A416V) 1: 54609779 17.3 1.19 × 10^–9^ 1.08

*TENM2* rs35769262 A/G 5: 168230416 43.0 1.44 × 10^–9^ 0.99

*TCN2* rs35915865 T/C (F89L) 22: 30612880 0.2 1.45 × 10^–9^ 0.94

*SDAD1* rs2273 C/T 4: 75968235 7.7 1.87 × 10^–9^ 1.03

*ABHD16B* rs150926976 G/A (V450M) 20: 63862888 0.4 2.10 × 10^–9^ 1.14

*SCN7A* rs6738031 A/C (I958M) 2: 166423412 29.6 4.39 × 10^–9^ 1.04

*ZNF543* rs8100491 G/A (R107Q) 19: 57327782 45.3 4.45 × 10^–9^ 1.05

*CAMTA1* rs138123146 A/G (T480A) 1: 7663985 0.4 4.50 × 10^–9^ 0.93

*ZC3HAV1* rs2297236 C/G (Q701E) 7: 139055291 40.2 6.16 × 10^–9^ 1.06

*VWF* rs1800377 C/T (V471I) 12: 6064267 15.4 6.19 × 10^–9^ 1.05

*SPDYA* rs6737027 C/T 2: 28847939 10.4 6.64 × 10^–9^ 1.13

rs9293471 A/G 5: 86664380 30.4 8.31 × 10^–9^ 0.93

*NUBP1* rs2233531 C/G (P39A) 16: 10744056 9.7 1.25 × 10^–8^ 1.01

*SQRDL* rs10643 G/A 15: 45689119 0.3 2.07 × 10^–8^ 1.50

*DUS2* rs202069030 G/C (R51S) 16: 68023050 0.4 4.72 × 10^–8^ 0.11

*ZNF85* rs56393308 C/G (T177R) 19: 20949044 1.6 5.55 × 10^–8^ 0.90

*GSTO1* rs201522765 C/T (P151L) 10: 104266154 0.3 6.09 × 10^–8^ 1.12

*C10orf120* rs74509433 C/G (S96R) 10: 122698453 13.4 7.17 × 10^–8^ 0.93

*PLOD2* rs1449444 G/T 3: 146073016 44.1 8.02 × 10^–8^ 1.00

*ACTR5* rs3752289 C/T (P580L) 20: 38771731 1.4 9.44 × 10^–8^ 0.91

*CDH23* rs200572025 G/A 10: 71812537 0.2 9.73 × 10^–8^ 1.34

*PRR16* rs17853861 C/A (P110T) 5: 120686122 6.9 1.10 × 10^–7^ 1.11

*TTN* rs55675869 C/T (V33366I) 2: 178537013 6.0 1.98 × 10^–7^ 1.02

*ZNF624* rs8065506 A/C (N135K) 17: 16624481 42.1 2.07 × 10^–7^ 0.96

rs439121 G/T 6: 33225090 35.0 2.31 × 10^–7^ 1.10

*ECM1* rs3737240 C/T (T130M) 1: 150510879 28.4 2.79 × 10^–7^ 0.92

*SEC14L4* rs9606739 G/C (G124R) 22: 30495307 2.8 3.55 × 10^–7^ 1.06

*LINC01476* rs8073039 A/C 17: 59443847 28.9 4.12 × 10^–7^ 1.10

ZYG11A rs480299 G/T (E76D) 1: 52854602 26.9 4.23 × 10^–7^ 0.95

*PPP1R3A* rs1800000 C/A (R883S) 7: 113878443 12.5 4.40 × 10^–7^ 0.92

*CMYA5* rs16877135 T/C (V1333A) 5: 79732763 49.3 5.16 × 10^–7^ 1.02

*FSCB* rs1959379 G/A (P409S) 14: 44505763 46.2 5.60 × 10^–7^ 0.94

*VWC2* rs10237193 A/C 7: 49796864 28.7 6.46 × 10^–7^ 1.08

*SPATC1L* rs113710653 C/T (E231K) 21: 46161921 1.9 7.01 × 10^–7^ 2.24

*USP5* rs143332941 G/A 12: 6859470 0.5 7.62 × 10^–7^ 1.22

*KCTD15* rs144406308 C/T (T197I) 19: 33811449 0.4 9.26 × 10^–7^ 1.22

*GLCE* rs12440300 A/G (M65V) 15: 69255999 3.3 1.04 × 10^–6^ 0.99

*KMT2B* rs76520998 C/G 19: 35739346 13.6 1.08 × 10^–6^ 1.09

*AKAP9* rs77447750 T/C (M3905T) 7: 92110149 0.3 1.11 × 10^–6^ 0.57

___________________________________________________________________________________

Allele frequencies were analyzed with Fisher’s exact test. ^a^Major allele/minor allele. MAF, minor allele frequency; OR, odds ratio.

**Supplementary Table 5.** Genotype distributions for single nucleotide polymorphisms (SNPs) significantly associated with myocardial infarction in the exome-wide association study.

____________________________________________________________________________________________________________

SNP Myocardial infarction H-W *P* Control H-W *P*

_________________________ ____________________________________ _____________________________________

rs75778351 C/G (L157V) *CC* *CG* *GG* *CC* *CG* *GG*

2434 (99.84) 4 (0.16) 0 (0) 1.0000 9188 (99.76) 22 (0.24) 0 (0) 1.0000

rs116911833 G/A (T80M) *GG* *GA* *AA*  *GG* *GA* *AA*

2350 (96.39) 87 (3.57) 1 (0.04) 0.5587 8823 (95.80) 383 (4.16) 4 (0.04) 1.0000

rs4662742 C/A *CC* *CA* *AA*  *CC* *CA* *AA*

1229 (50.41) 1027 (42.13) 182 (7.46) 0.1119 4683 (50.85) 3788 (41.14) 738 (8.01) 0.4749

rs41289932 C/G (N310K) *CC* *CG* *GG* *CC* *CG* *GG*

2206 (90.48) 221 (9.07) 11 (0.45) 0.0492 8371 (90.90) 816 (8.86) 22 (0.24) 0.6388

rs11726613 A/C *AA* *AC* *CC*  *AA* *AC* *CC*

979 (40.16) 1139 (46.72) 320 (13.12) 0.7266 3578 (38.85) 4309 (46.79) 1323 (14.36) 0.6575

rs117417589 A/G (K406E) *AA* *AG* *GG*  *AA* *AG* *GG*

2333 (95.69) 104 (4.27) 1 (0.04) 1.0000 8867 (96.33) 337 (3.66) 1 (0.01) 0.3786

rs202103723 C/A (P511Q) *CC* *CA* *AA*  *CC* *CA* *AA*

2405 (98.65) 32 (1.31) 1 (0.04) 0.1094 9161 (99.47) 49 (0.53) 0 (0) 1.0000

rs56188613 C/A (G451V) *CC* *CA* *AA*  *CC* *CA* *AA*

2395 (98.24) 43 (1.76) 0 (0) 1.0000 9037 (98.12) 171 (1.86) 2 (0.02) 0.2002

rs6557316 G/A *GG* *GA* *AA*  *GG* *GA* *AA*

705 (28.92) 1149 (47.13) 584 (23.95) 0.0065 2505 (27.20) 4587 (49.80) 2118 (23.00) 0.8347

rs3135365 T/G *TT* *TG* *GG*  *TT* *TG* *GG*

1622 (66.61) 721 (29.61) 92 (3.78) 0.2843 5985 (64.98) 2886 (31.34) 339 (3.68) 0.7135

rs193164904 A/G (I534V) *AA* *AG* *GG*  *AA* *AG* *GG*

2431 (99.71) 7 (0.29) 0 (0) 1.0000 9180 (99.67) 30 (0.33) 0 (0) 1.0000

rs11624336 G/A *GG* *GA* *AA*  *GG* *GA* *AA*

1804 (74.00) 565 (23.17) 69 (2.83) 0.0039 6655 (72.26) 2334 (25.34) 221 (2.40) 0.3288

rs3204853 C/A (R31L) *CC* *CA* *AA*  *CC* *CA* *AA*

2361 (96.84) 77 (3.16) 0 (0) 1.0000 8945 (97.13) 260 (2.82) 5 (0.05) 0.0475

rs41267732 A/T (M416L) *AA* *AT* *TT*  *AA* *AT* *TT*

2398 (98.36) 39 (1.60) 1 (0.04) 0.1560 9031 (98.09) 176 (1.91) 0 (0) 1.0000

rs2925979 G/A *GG* *GA* *AA*  *GG* *GA* *AA*

1059 (43.44) 1101 (45.16) 278 (11.40) 0.7865 4039 (43.86) 4119 (44.73) 1051 (11.41) 0.9814

rs6965750 G/A *GG* *GA* *AA*  *GG* *GA* *AA*

2229 (91.43) 206 (8.45) 3 (0.12) 0.6249 8415 (91.37) 770 (8.36) 25 (0.27) 0.1094

rs181428774 G/T (L59M) *GG* *GT* *TT*  *GG* *GT* *TT*

2422 (99.34) 16 (0.66) 0 (0) 1.0000 9140 (99.24) 69 (0.75) 1 (0.01) 0.1267

rs9324700 A/G (F686S) *AA* *AG* *GG*  *AA* *AG* *GG*

1276 (52.34) 951 (39.01) 211 (8.65) 0.0797 4553 (49.45) 3803 (41.30) 852 (9.25) 0.1565

rs7758229 G/T *GG* *GT* *TT*  *GG* *GT* *TT*

1393 (57.18) 904 (37.11) 139 (5.71) 0.6593 5318 (57.77) 3343 (36.32) 544 (5.91) 0.5493

rs2010834 A/C (F254C) *AA* *AC* *CC*  *AA* *AC* *CC*

1411 (57.88) 879 (36.05) 148 (6.07) 0.4719 5206 (56.52) 3459 (37.56) 545 (5.92) 0.3554

rs1551888 T/C (N52S) *TT* *TC* *CC*  *TT* *TC* *CC*

7 (0.29) 218 (8.94) 2213 (90.77) 0.4977 8284 (89.99) 901 (9.79) 20 (0.22) 0.4515

rs56231757 C/T (S1901N) *CC* *CT* *TT*  *CC* *CT* *TT*

1921 (78.83) 489 (20.06) 27 (1.11) 0.6084 7396 (80.30) 1717 (18.65) 97 (1.05) 0.8665

rs1563631 C/T *CC* *CT* *TT*  *CC* *CT* *TT*

2058 (84.41) 363 (14.89) 17 (0.70) 0.7864 7716 (83.78) 1422 (15.44) 72 (0.78) 0.4611

rs79742527 G/A *GG* *GA* *AA*  *GG* *GA* *AA*

1797 (73.77) 593 (24.34) 46 (1.89) 0.8013 6863 (74.52) 2173 (23.59) 174 (1.89) 0.8947

rs148608964 C/T (P1241L) *CC* *CT* *TT*  *CC* *CT* *TT*

2373 (97.34) 62 (2.54) 3 (1.2) 0.0106 8925 (96.91) 282 (3.06) 3 (0.03) 0.4923

rs188212047 G/T (L212F) *GG* *GT* *TT*  *GG* *GT* *TT*

2359 (97.12) 70 (2.88) 0 (0) 1.0000 9047 (98.84) 106 (1.16) 0 (0) 1.0000

rs143833298 G/A (R830Q) *GG* *GA* *AA*  *GG* *GA* *AA*

2406 (98.69) 32 (1.31) 0 (0) 1.0000 9053 (98.30) 156 (1.69) 1 (0.01) 0.4930

rs1052878 C/T (P922L) *CC* *CT* *TT*  *CC* *CT* *TT*

2238 (91.80) 194 (7.96) 6 (0.24) 0.4447 8305 (90.17) 883 (9.59) 22 (0.24) 0.9130

rs201169529 A/G (Y790C) *AA* *AG* *GG*  *AA* *AG* *GG*

2406 (98.69) 32 (1.31) 0 (0) 1.0000 9104 (98.85) 105 (1.14) 1 (0.01) 0.2663

rs1265110 G/A *GG* *GA* *AA*  *GG* *GA* *AA*

1282 (52.58) 954 (39.13) 202 (8.29) 1.0000 4414 (47.94) 3943 (42.82) 851 (9.24) 0.3474

rs77336780 C/G (A304G) *CC* *CG* *GG* *CC* *CG* *GG*

1583 (64.93) 768 (31.50) 87 (3.57) 0.6493 5816 (63.16) 2975 (32.30) 418 (4.54) 0.1356

rs1480347 G/A *GG* *GA* *AA*  *GG* *GA* *AA*

1707 (70.02) 662 (27.15) 69 (2.83) 0.6055 6285 (68.24) 2637 (28.63) 288 (3.13) 0.5628

rs11641035 G/A *GG* *GA* *AA*  *GG* *GA* *AA*

1764 (72.39) 622 (25.52) 51 (2.09) 0.7485 6720 (72.97) 2286 (24.83) 203 (2.20) 0.5875

rs80143472 G/C (E952D) *GG* *GC* *CC*  *GG* *GC* *CC*

2094 (85.89) 329 (13.50) 15 (0.61) 0.5529 7939 (86.22) 1215 (13.19) 54 (0.59) 0.3093

rs2293012 T/G (M9L) *TT* *TG* *GG*  *TT* *TG* *GG*

1173 (48.13) 1030 (42.27) 234 (9.60) 0.7391 4445 (48.30) 2865 (42.00) 893 (9.70) 0.2116

rs2231599 A/G (S492G) *AA* *AG* *GG*  *AA* *AG* *GG*

2380 (97.62) 58 (2.38) 0 (0) 1.0000 9015 (97.88) 193 (2.10) 2 (0.02) 0.2821

rs12146904 G/A *GG* *GA* *AA*  *GG* *GA* *AA*

752 (30.84) 1204 (49.39) 482 (19.77) 1.0000 2813 (30.55) 4580 (49.73) 1816 (19.72) 0.5409

rs138559558 G/A (R289C) *GG* *GA* *AA*  *GG* *GA* *AA*

2376 (97.46) 60 (2.46) 2 (0.08) 0.0631 8985 (97.56) 225 (2.44) 0 (0) 0.6490

rs2305296 A/G (I36V) *AA* *AG* *GG*  *AA* *AG* *GG*

2335 (95.78) 102 (4.18) 1 (0.04) 1.0000 8843 (96.02) 363 (3.94) 4 (0.04) 0.7895

rs56198402 G/A (V454I) *GG* *GA* *AA*  *GG* *GA* *AA*

2405 (98.69) 32 (1.31) 0 (0) 1.0000 9092 (98.72) 118 (1.28) 0 (0) 1.0000

rs36022742 C/T (R3K) *CC* *CT* *TT*  *CC* *CT* *TT*

2361 (96.84) 76 (3.12) 1 (0.04) 0.4652 8926 (96.92) 282 (3.06) 2 (0.02) 1.0000

rs182666831 A/T (S48C) *AA* *AT* *TT*  *AA* *AT* *TT*

2432 (99.75) 6 (0.25) 0 (0) 1.0000 9180 (99.67) 30 (0.33) 0 (0) 1.0000

rs138695721 A/C (V139G) *AA* *AC* *CC*  *AA* *AC* *CC*

2417 (99.18) 20 (0.82) 0 (0) 1.0000 9094 (98.74) 116 (1.26) 0 (0) 1.0000

rs41314525 C/A (R85L) *CC* *CA* *AA*  *CC* *CA* *AA*

898 (36.83) 1151 (47.21) 389 (15.96) 0.5255 3415 (37.08) 4321 (46.92) 1474 (16.00) 0.0849

rs11007350 C/T *CC* *CT* *TT*  *CC* *CT* *TT*

1473 (60.44) 845 (34.68) 119 (4.88) 0.9068 5312 (57.68) 3347 (36.34) 551 (5.98) 0.4425

rs7752978 A/G *AA* *AG* *GG*  *AA* *AG* *GG*

685 (28.11) 1204 (49.40) 548 (22.49) 0.6844 2573 (27.94) 4571 (49.64) 2065 (22.42) 0.6912

rs1910795 C/T *CC* *CT* *TT*  *CC* *CT* *TT*

628 (25.76) 1193 (48.93) 617 (25.31) 0.2925 2315 (25.16) 4509 (49.00) 2378 (25.84) 0.0551

rs10937405 C/T *CC* *CT* *TT*  *CC* *CT* *TT*

1171 (48.03) 1027 (42.13) 240 (9.84) 0.5068 4305 (46.74) 3948 (42.87) 957 (10.39) 0.2490

rs34837068 A/G (V125A) *AA* *AG* *GG*  *AA* *AG* *GG*

2211 (90.84) 212 (8.71) 11 (0.45) 0.0248 8298 (90.12) 887 (9.63) 23 (0.25) 1.0000

rs117378923 G/A (V144M) *GG* *GA* *AA*  *GG* *GA* *AA*

2415 (99.06) 23 (0.94) 0 (0) 1.0000 9101 (98.82) 109 (1.18) 0 (0) 1.0000

rs17847749 G/C (W691C) *GG* *GC* *CC*  *GG* *GC* *CC*

2412 (99.06) 22 (0.90) 1 (0.04) 0.0554 9132 (99.27) 66 (0.72) 1 (0.01) 0.1169

rs1057066 G/C (G187A) *GG* *GC* *CC*  *GG* *GC* *CC*

917 (49.04) 789 (42.19) 164 (8.77) 0.7827 4584 (49.77) 3831 (41.60) 795 (8.63) 0.9002

rs45476795 G/T (T651N) *GG* *GT* *TT*  *GG* *GT* *TT*

2401 (98.48) 37 (1.52) 0 (0) 1.0000 9058 (98.35) 149 (1.62) 3 (0.03) 0.0269

rs114335521 A/G (Y203C) *AA* *AG* *GG*  *AA* *AG* *GG*

2412 (98.93) 26 (1.07) 0 (0) 1.0000 9111 (98.93) 99 (1.07) 0 (0) 1.0000

rs586088 A/T (T190S) *AA* *AT* *TT*  *AA* *AT* *TT*

1142 (46.84) 1055 (43.27) 241 (9.89) 0.9253 4349 (47.22) 3913 (42.49) 948 (10.29) 0.1225

rs13223723 T/C *TT* *TC* *CC*  *TT* *TC* *CC*

1824 (75.65) 540 (22.40) 47 (1.95) 0.3274 6976 (76.28) 2037 (22.28) 132 (1.44) 0.2348

rs904763 G/T (P685H) *GG* *GT* *TT*  *GG* *GT* *TT*

1149 (47.13) 1040 (42.66) 249 (10.21) 0.5423 4286 (46.54) 3956 (42.96) 967 (10.50) 0.2310

rs12348881 T/G *TT* *TG* *GG*  *TT* *TG* *GG*

2271 (93.15) 166 (6.81) 1 (0.04) 0.3658 8604 (93.47) 594 (6.45) 7 (0.08) 0.4133

rs201986662 T/C (I472M) *TT* *TC* *CC*  *TT* *TC* *CC*

2419 (99.22) 19 (0.78) 0 (0) 1.0000 9174 (99.61) 36 (0.39) 0 (0) 1.0000

rs10794531 C/T (R53H) *CC* *CT* *TT*  *CC* *CT* *TT*

941 (38.60) 1121 (45.98) 376 (15.42) 0.1707 3635 (39.47) 4277 (46.44) 1298 (14.09) 0.4899

rs143953605 C/T (V55I) *CC* *CT* *TT*  *CC* *CT* *TT*

2403 (98.56) 35 (1.44) 0 (0) 1.0000 9051 (98.27) 158 (1.72) 1 (0.01) 0.5017

rs1859962 T/G *TT* *TG* *GG*  *TT* *TG* *GG*

1342 (55.04) 950 (38.97) 146 (5.99) 0.2009 5045 (54.78) 3545 (38.49) 620 (6.73) 0.9568

rs2634041 C/T (G101S) *CC* *CT* *TT*  *CC* *CT* *TT*

1376 (56.51) 899 (36.92) 160 (6.57) 0.4182 5267 (57.19) 3401 (36.93) 542 (5.88) 0.8431

rs200536426 G/A (D4519N) *GG* *GA* *AA*  *GG* *GA* *AA*

2421 (99.30) 17 (0.67) 0 (0) 1.0000 9111 (98.93) 99 (1.07) 0 (0) 1.0000

rs9293688 T/C *TT* *TC* *CC*  *TT* *TC* *CC*

1062 (43.60) 1077 (44.21) 297 (12.19) 0.3454 3953 (42.92) 4177 (45.36) 1079 (11.72) 0.6442

rs192115090 G/C (A2210P) *GG* *GC* *CC*  *GG* *GC* *CC*

2389 (97.99) 49 (2.01) 0 (0) 1.0000 9057 (98.34) 151 (1.64) 2 (0.02) 0.1369

rs1233397 C/T *CC* *CT* *TT*  *CC* *CT* *TT*

656 (26.91) 1181 (48.44) 601 (24.65) 0.1337 2552 (27.71) 4546 (49.56) 2112 (22.93) 0.3160

rs2745400 G/A *GG* *GA* *AA*  *GG* *GA* *AA*

716 (29.38) 1201 (49.28) 520 (21.34) 0.7136 2735 (29.70) 4441 (48.22) 2033 (22.08) 0.0044

rs9258102 T/C *TT* *TC* *CC*  *TT* *TC* *CC*

1894 (77.72) 515 (21.13) 28 (1.15) 0.3274 7459 (81.03) 1653 (17.96) 93 (1.01) 0.8621

rs2643184 A/G *AA* *AG* *GG*  *AA* *AG* *GG*

861 (35.33) 1170 (48.01) 406 (16.66) 0.8012 3174 (34.49) 4451 (48.36) 1578 (17.15) 0.7965

rs74491133 C/T *CC* *CT* *TT*  *CC* *CT* *TT*

2367 (97.09) 71 (2.91) 0 (0) 1.0000 8964 (97.34) 243 (2.64) 2 (0.02) 0.6816

rs79060400 T/C *TT* *TC* *CC*  *TT* *TC* *CC*

1763 (73.28) 605 (25.14) 38 (1.58) 0.0934 5265 (75.28) 1585 (22.66) 144 (2.06) 0.0564

rs200867550 C/T (V874I) *CC* *CT* *TT*  *CC* *CT* *TT*

2436 (99.92) 2 (0.08) 0 (0) 1.0000 9176 (99.63) 34 (0.37) 0 (0) 1.0000

rs16927649 G/A (R425Q) *GG* *GA* *AA*  *GG* *GA* *AA*

2371 (97.25) 65 (2.67) 2 (0.08) 0.0823 8975 (97.45) 232 (2.52) 3 (0.03) 0.1974

rs56288451 C/T (P903S) *CC* *CT* *TT*  *CC* *CT* *TT*

1774 (72.79) 607 (24.91) 56 (2.30) 0.6289 6759 (73.39) 2247 (24.40) 204 (2.21) 0.2909

rs1368883 G/A (A416V) *GG* *GA* *AA*  *GG* *GA* *AA*

1639 (67.34) 720 (29.58) 75 (3.08) 0.7823 6357 (69.27) 2568 (27.98) 252 (2.75) 0.7361

rs35769262 A/G *AA* *AG* *GG*  *AA* *AG* *GG*

807 (33.10) 1179 (48.36) 452 (18.54) 0.5624 3048 (33.09) 4423 (48.03) 1739 (18.88) 0.0584

rs35915865 T/C (F89L) *TT* *TC* *CC*  *TT* *TC* *CC*

2428 (99.63) 9 (0.37) 0 (0) 1.0000 9174 (99.61) 36 (0.39) 0 (0) 1.0000

rs2273 C/T *CC* *CT* *TT*  *CC* *CT* *TT*

2061 (84.57) 361 (14.81) 15 (0.62) 1.0000 7839 (85.12) 1305 (14.17) 65 (0.71) 0.1913

rs150926976 G/A (V450M) *GG* *GA* *AA*  *GG* *GA* *AA*

2412 (98.93) 26 (1.07) 0 (0) 1.0000 9124 (99.07) 86 (0.93) 0 (0) 1.0000

rs6738031 A/C (I958M) *AA* *AC* *CC*  *AA* *AC* *CC*

1185 (48.60) 1019 (41.80) 234 (9.60) 0.5035 4587 (49.81) 3790 (41.15) 833 (9.04) 0.2115

rs8100491 G/A (R107Q) *GG* *GA* *AA*  *GG* *GA* *AA*

691 (28.36) 1229 (50.43) 517 (21.21) 0.5148 2751 (29.87) 4582 (49.75) 1877 (20.38) 0.7051

rs138123146 A/G (T480A) *AA* *AG* *GG*  *AA* *AG* *GG*

2417 (99.14) 21 (0.86) 0 (0) 1.0000 9125 (99.08) 85 (0.92) 0 (0) 1.0000

rs2297236 C/G (Q701E) *CC* *CG* *GG* *CC* *CG* *GG*

869 (35.66) 1145 (46.98) 423 (17.36) 0.1797 3405 (36.99) 4322 (46.95) 1479 (16.06) 0.0850

rs1800377 C/T (V471I) *CC* *CT* *TT*  *CC* *CT* *TT*

1734 (71.15) 641 (26.30) 62 (2.55) 0.7595 6644 (72.14) 2357 (25.59) 209 (2.27) 1.0000

rs6737027 C/T *CC* *CT* *TT*  *CC* *CT* *TT*

1929 (79.12) 479 (19.65) 30 (1.23) 0.9182 7487 (81.30) 1613 (17.52) 109 (1.18) 0.0414

rs9293471 A/G *AA* *AG* *GG*  *AA* *AG* *GG*

1220 (50.04) 1014 (41.59) 204 (8.37) 0.7688 4456 (48.39) 3872 (42.04) 881 (9.57) 0.3511

rs2233531 C/G (P39A) *CC* *CG* *GG* *CC* *CG* *GG*

1996 (81.87) 415 (17.02) 27 (1.11) 0.2941 7545 (81.96) 1574 (17.10) 87 (0.94) 0.6274

rs10643 G/A *GG* *GA* *AA*  *GG* *GA* *AA*

2417 (99.14) 21 (0.86) 0 (0) 1.0000 9157 (99.43) 53 (0.57) 0 (0) 1.0000

rs202069030 G/C (R51S) *GG* *GC* *CC*  *GG* *GC* *CC*

2407 (99.88) 3 (0.12) 0 (0) 1.0000 9039 (98.83) 107 (1.17) 0 (0) 1.0000

rs56393308 C/G (T177R) *CC* *CG* *GG* *CC* *CG* *GG*

2366 (97.13) 70 (2.87) 0 (0) 1.0000 8916 (96.86) 284 (3.09) 5 (0.05) 0.0852

rs201522765 C/T (P151L) *CC* *CT* *TT*  *CC* *CT* *TT*

2420 (99.26) 18 (0.74) 0 (0) 1.0000 9148 (99.34) 61 (0.66) 0 (0) 1.0000

rs74509433 C/G (S96R) *CC* *CG* *GG* *CC* *CG* *GG*

1432 (76.58) 404 (21.60) 34 (1.82) 0.4007 6889 (74.84) 2153 (23.39) 163 (1.77) 0.7543

rs1449444 G/T *GG* *GT* *TT*  *GG* *GT* *TT*

765 (31.38) 1196 (49.06) 477 (19.56) 0.8054 2908 (31.60) 4471 (48.59) 1823 (19.81) 0.1629

rs3752289 C/T (P580L) *CC* *CT* *TT*  *CC* *CT* *TT*

2376 (97.46) 62 (2.54) 0 (0) 1.0000 8959 (97.28) 246 (2.67) 5 (0.05) 0.0323

rs200572025 G/A *GG* *GA* *AA*  *GG* *GA* *AA*

2426 (99.55) 11 (0.45) 0 (0) 1.0000 9178 (99.66) 31 (0.34) 0 (0) 1.0000

rs17853861 C/A (P110T) *CC* *CA* *AA*  *CC* *CA* *AA*

2089 (85.72) 329 (13.50) 19 (0.78) 0.1434 8004 (86.91) 1157 (12.56) 49 (0.53) 0.2867

rs55675869 C/T (V33366I) *CC* *CT* *TT*  *CC* *CT* *TT*

2151 (88.23) 276 (11.32) 11 (0.45) 0.4786 8143 (88.41) 1034 (11.23) 33 (0.36) 0.9262

rs8065506 A/C (N135K) *AA* *AC* *CC*  *AA* *AC* *CC*

845 (34.66) 1157 (47.46) 436 (17.88) 0.2604 3043 (33.04) 4489 (48.74) 1678 (18.22) 0.7493

rs439121 G/T *GG* *GT* *TT*  *GG* *GT* *TT*

988 (40.52) 1100 (45.12) 350 (14.36) 0.1280 3931 (42.69) 4147 (45.03) 1131 (12.28) 0.4625

rs3737240 C/T (T130M) *CC* *CT* *TT*  *CC* *CT* *TT*

1298 (53.26) 960 (39.39) 179 (7.35) 0.9183 4689 (50.91) 3768 (40.91) 753 (8.18) 0.9390

rs9606739 G/C (G124R) *GG* *GC* *CC*  *GG* *GC* *CC*

2296 (94.18) 141 (5.78) 1 (0.04) 0.7220 8700 (94.46) 508 (5.52) 2 (0.02) 0.0506

rs8073039 A/C *AA* *AC* *CC*  *AA* *AC* *CC*

1181 (48.44) 1040 (42.66) 217 (8.90) 0.5976 4712 (51.17) 3777 (41.02) 719 (7.81) 0.3294

rs480299 G/T (E76D) *GG* *GT* *TT*  *GG* *GT* *TT*

1332 (54.63) 943 (38.68) 163 (6.69) 0.8746 4901 (53.21) 3625 (39.36) 684 (7.43) 0.7120

rs1800000 C/A (R883S) *CC* *CA* *AA*  *CC* *CA* *AA*

1900 (77.93) 499 (20.47) 39 (1.60) 0.3322 7002 (76.03) 2067 (22.44) 141 (1.53) 0.4263

rs16877135 T/C (V1333A) *TT* *TC* *CC*  *TT* *TC* *CC*

614 (25.18) 1217 (49.92) 607 (24.90) 0.9355 2413 (26.21) 4500 (48.87) 2295 (24.92) 0.0318

rs1959379 G/A (P409S) *GG* *GA* *AA*  *GG* *GA* *AA*

756 (31.01) 1167 (47.87) 515 (21.12) 0.1019 2641 (28.72) 4530 (49.27) 2024 (22.01) 0.3248

rs10237193 A/C *AA* *AC* *CC*  *AA* *AC* *CC*

1219 (50.02) 989 (40.58) 229 (9.40) 0.1742 4755 (51.63) 3724 (40.44) 730 (7.93) 0.9794

rs113710653 C/T (E231K) *CC* *CT* *TT*  *CC* *CT* *TT*

1109 (94.79) 61 (5.21) 0 (0) 1.0000 6918 (97.69) 161 (2.27) 3 (0.04) 0.0742

rs143332941 G/A *GG* *GA* *AA*  *GG* *GA* *AA*

2411 (98.89) 27 (1.11) 0 (0) 1.0000 9126 (99.09) 84 (0.91) 0 (0) 1.0000

rs144406308 C/T (T197I) *CC* *CT* *TT*  *CC* *CT* *TT*

2415 (99.06) 23 (0.94) 0 (0) 1.0000 9141 (99.25) 67 (0.73) 2 (0.02) 0.0079

rs12440300 A/G (M65V) *AA* *AG* *GG*  *AA* *AG* *GG*

2278 (93.44) 160 (6.56) 0 (0) 0.1113 8616 (93.55) 580 (6.30) 14 (0.15) 0.1897

rs76520998 C/G *CC* *CG* *GG* *CC* *CG* *GG*

1793 (73.57) 589 (24.17) 55 (2.26) 0.4100 6919 (75.19) 2117 (23.01) 166 (1.80) 0.7963

rs77447750 T/C (M3905T) *TT* *TC* *CC*  *TT* *TC* *CC*

2430 (99.67) 8 (0.33) 0 (0) 1.0000 9158 (99.44) 51 (0.55) 1 (0.01) 0.0723

____________________________________________________________________________________________________________

Data are numbers of subjects (percentages). H-W *P*, *P* value for Hardy-Weinberg equilibrium.

**Supplementary Table 6.** Relation of single nucleotide polymorphisms (SNPs) to myocardial infarction as determined by multivariable logistic regression analysis.

__________________________________________________________________________________________________________________________________

SNP Dominant Recessive Additive 1 Additive 2

____________________ ____________________ ____________________ ____________________

*P* OR (95% CI) *P* OR (95% CI) *P* OR (95% CI) *P* OR (95% CI)

__________________________________________________________________________________________________________________________________

rs75778351 C/G (L157V) 0.2879 ND 0.2879 ND

rs116911833 G/A (T80M) 0.2227 0.9926 0.2204 0.9882

rs4662742 C/A 0.6069 0.2038 0.3541 0.3139

rs41289932 C/G (N310K) 0.4071 0.3300 0.5066 0.3240

rs11726613 A/C 0.3431 0.4800 0.4460 0.3476

rs117417589 A/G (K406E) 0.4783 0.8282 0.4761 0.8285

rs202103723 C/A (P511Q) 0.0006 2.68 (1.54–4.58) 0.0448 >100 (ND) 0.0015 2.54 (1.44–4.37) 0.0443 >100 (ND)

rs56188613 C/A (G451V) 0.6491 0.5261 0.6767 0.5258

rs6557316 G/A 0.0606 0.3822 0.0206 0.86 (0.75–0.98) 0.6187

rs3135365 T/G 0.2161 0.6796 0.1614 0.8188

rs193164904 A/G (I534V) 0.8901 ND 0.8901 ND

rs11624336 G/A 0.3954 0.0923 0.1764 0.1222

rs3204853 C/A (R31L) 0.2538 0.3742 0.2287 0.3755

rs41267732 A/T (M416L) 0.3773 0.3218 0.3313 0.3224

rs2925979 G/A 0.9341 0.8514 0.9803 0.8350

rs6965750 G/A 0.3240 0.5700 0.2774 0.5808

rs181428774 G/T (L59M) 0.8195 0.7546 0.8314 0.7546

rs9324700 A/G (F686S) 0.0788 0.5913 0.0941 0.3354

rs7758229 G/T 0.7513 0.4280 0.5674 0.5017

rs2010834 A/C (F254C) 0.3537 0.2060 0.1769 0.3244

rs1551888 T/C (N52S) 0.1290 0.6917 0.1092 0.7120

rs56231757 C/T (S1901N) 0.0510 0.6113 0.0592 0.5432

rs1563631 C/T 0.5668 0.2564 0.7274 0.2512

rs79742527 G/A 0.1395 0.8291 0.1417 0.7402

rs148608964 C/T (P1241L) 0.6272 0.1843 0.4674 0.1853

rs188212047 G/T (L212F) **4.84 × 10^-8^** 2.94 (2.02–4.24) ND **4.84 × 10^-8^** 2.94 (2.02–4.24) ND

rs143833298 G/A (R830Q) 0.4623 0.7736 0.4674 0.7733

rs1052878 C/T (P922L) 0.0398 0.82 (0.67–0.99) 0.6591 0.0307 0.81 (0.66–0.98) 0.6862

rs201169529 A/G (Y790C) 0.7090 0.5877 0.6834 0.5879

rs1265110 G/A 0.0005 0.82 (0.74–0.92) 0.0719 0.0020 0.83 (0.74–0.94) 0.0114 0.77 (0.62–0.94)

rs77336780 C/G (A304G) 0.1087 0.0154 0.71 (0.52–0.94) 0.3317 0.0112 0.69 (0.51–0.92)

rs1480347 G/A 0.0688 0.3164 0.1061 0.2425

rs11641035 G/A 0.6118 0.7487 0.6582 0.7204

rs80143472 G/C (E952D) 0.7763 0.2234 0.9640 0.2231

rs2293012 T/G (M9L) 0.3573 0.8277 0.3694 0.6462

rs2231599 A/G (S492G) 0.5927 0.6154 0.5767 0.6158

rs12146904 G/A 0.7255 0.6377 0.8367 0.6089

rs138559558 G/A (R289C) 0.9473 0.0017 >100 (ND) 0.7886 0.0017 >100 (ND)

rs2305296 A/G (I36V) 0.6854 0.2426 0.6241 0.2432

rs56198402 G/A (V454I) 0.8264 ND 0.8264 ND

rs36022742 C/T (R3K) 0.8119 0.9480 0.8167 0.9474

rs182666831 A/T (S48C) 0.6805 ND 0.6805 ND

rs138695721 A/C (V139G) 0.0863 ND 0.0863 ND

rs41314525 C/A (R85L) 0.6844 0.9917 0.6691 0.8528

rs11007350 C/T 0.0067 0.86 (0.77–0.96) 0.0394 0.77 (0.60–0.99) 0.0270 0.88 (0.78–0.99) 0.0155 0.73 (0.57–0.94)

rs7752978 A/G 0.7256 0.6154 0.8468 0.5974

rs1910795 C/T 0.9626 0.7796 0.9583 0.8419

rs10937405 C/T 0.7878 0.5430 0.6252 0.6599

rs34837068 A/G (V125A) 0.4521 0.0623 0.2615 0.0656

rs117378923 G/A (V144M) 0.3070 ND 0.3070 ND

rs17847749 G/C (W691C) 0.3362 0.3180 0.4319 0.3174

rs1057066 G/C (G187A) 0.5716 0.7034 0.6368 0.6235

rs45476795 G/T (T651N) 0.2722 0.2936 0.3185 0.2928

rs114335521 A/G (Y203C) 0.2426 ND 0.2426 ND

rs586088 A/T (T190S) 0.5723 0.8967 0.5226 0.9507

rs13223723 T/C 0.3243 0.3140 0.4403 0.2900

rs904763 G/T (P685H) 0.4555 0.6745 0.5180 0.5539

rs12348881 T/G 0.5169 0.8214 0.5007 0.8251

rs201986662 T/C (I472M) 0.0509 ND 0.0509 ND

rs10794531 C/T (R53H) 0.7257 0.5434 0.8725 0.5335

rs143953605 C/T (V55I) 0.3588 0.4348 0.3873 0.4343

rs1859962 T/G 0.8974 0.5357 0.7451 0.5905

rs2634041 C/T (G101S) 0.2013 0.4615 0.2673 0.3468

rs200536426 G/A (D4519N) 0.1301 ND 0.1301 ND

rs9293688 T/C 0.6744 0.2078 0.9893 0.2335

rs192115090 G/C (A2210P) 0.2792 0.4061 0.2516 0.4070

rs1233397 C/T 0.3192 0.2024 0.5500 0.1639

rs2745400 G/A 0.4612 0.5834 0.5583 0.4415

rs9258102 T/C 0.0010 1.25 (1.10–1.43) 0.7389 0.0010 1.26 (1.10–1.45) 0.6189

rs2643184 A/G 0.9843 0.9875 0.9790 0.9981

rs74491133 C/T 0.6270 0.6354 0.6136 0.6358

rs79060400 T/C 0.0675 0.1521 0.0260 1.16 (1.02–1.32) 0.2096

rs200867550 C/T (V874I) 0.0083 0.13 (0.01–0.65) ND 0.0083 0.13 (0.01–0.65) ND

rs16927649 G/A (R425Q) 0.1499 0.2485 0.2018 0.2646

rs56288451 C/T (P903S) 0.1866 0.2810 0.2757 0.2429

rs1368883 G/A (A416V) 0.0468 1.13 (1.00–1.27) 0.4100 0.0655 0.3103

rs35769262 A/G 0.7470 0.9928 0.7341 0.8694

rs35915865 T/C (F89L) 0.7776 ND 0.7776 ND

rs2273 C/T 0.7457 0.2139 0.9317 0.2131

rs150926976 G/A (V450M) 0.5167 ND 0.5167 ND

rs6738031 A/C (I958M) 0.2617 0.9909 0.2390 0.7405

rs8100491 G/A (R107Q) 0.2376 0.5352 0.3003 0.2870

rs138123146 A/G (T480A) 0.5018 ND 0.5018 ND

rs2297236 C/G (Q701E) 0.0832 0.6904 0.0898 0.2853

rs1800377 C/T (V471I) 0.8556 0.2787 0.6372 0.3008

rs6737027 C/T 0.0461 1.15 (1.00–1.32) 0.8115 0.0350 1.16 (1.01–1.34) 0.9017

rs9293471 A/G 0.0344 0.89 (0.80–0.99) 0.0244 0.80 (0.66–0.97) 0.1373 0.0100 0.77 (0.63–0.94)

rs2233531 C/G (P39A) 0.2431 0.9449 0.2385 0.9045

rs10643 G/A 0.1489 ND 0.1489 ND

rs202069030 G/C (R51S) 0.0466 0.36 (0.09–0.99) ND 0.0466 0.36 (0.09–0.99) ND

rs56393308 C/G (T177R) 0.5025 0.2723 0.5573 0.2716

rs201522765 C/T (P151L) 0.6245 ND 0.6245 ND

rs74509433 C/G (S96R) 0.1106 0.3935 0.0653 0.4720

rs1449444 G/T 0.9834 0.9530 0.9659 0.9754

rs3752289 C/T (P580L) 0.4786 0.2050 0.5523 0.2044

rs200572025 G/A 0.0623 ND 0.0623 ND

rs17853861 C/A (P110T) 0.0916 0.1811 0.1477 0.1670

rs55675869 C/T (V33366I) 0.5235 0.8808 0.4993 0.8937

rs8065506 A/C (N135K) 0.5492 0.5024 0.6920 0.4370

rs439121 G/T 0.1481 0.0060 1.25 (1.07–1.46) 0.5685 0.0056 1.27 (1.07–1.51)

rs3737240 C/T (T130M) 0.0243 0.88 (0.79–0.98) 0.2088 0.0496 0.89 (0.79–1.00) 0.0911

rs9606739 G/C (G124R) 0.6333 0.2011 0.6955 0.2006

rs8073039 A/C 0.1930 0.1057 0.3963 0.0751

rs480299 G/T (E76D) 0.1204 0.0848 0.2769 0.0543

rs1800000 C/A (R883S) 0.2224 0.4088 0.1473 0.4663

rs16877135 T/C (V1333A) 0.4040 0.4897 0.2555 0.9329

rs1959379 G/A (P409S) 0.0605 0.1198 0.1538 0.0389 0.85 (0.43–0.99)

rs10237193 A/C 0.2052 0.3200 0.3148 0.2241

rs113710653 C/T (E231K) 0.3424 0.5251 0.3649 0.5242

rs143332941 G/A 0.1596 ND 0.1596 ND

rs144406308 C/T (T197I) 0.1466 0.4968 0.1263 0.4977

rs12440300 A/G (M65V) 0.3563 0.0143 <0.01 (ND) 0.5018 0.0141 <0.01 (0–0.58)

rs76520998 C/G 0.4554 0.3528 0.5890 0.3329

rs77447750 T/C (M3905T) 0.0806 0.5295 0.0914 0.5286

___________________________________________________________________________________________________________________________________

Multivariable logistic regression analysis was performed with adjustment for age, sex, and the prevalence of hypertension, diabetes mellitus, and dyslipidemia. Based on Bonferroni’s correction, *P* values of <1.10 × 10^–4^ (0.05/456) were considered statistically significant and are shown in bold. OR, odds ratio; CI, confidence interval; ND, not determined.

**Supplementary Table 8.** Relation of chromosomal loci, genes, and single nucleotide polymorphisms (SNPs) identified in the present study to phenotypes previously examined in genome-wide association studies.

| Gene  (chr. locus) | SNP | Nucleotide  (amino acid)  substitution | Previously examined phenotypes |
| --- | --- | --- | --- |
| Related to CAD | | | |
| *WDR66* | rs58098972 | A/G | [Urinary metabolites](http://www.ebi.ac.uk/gwas/search?query=Urinary%20metabolites) (PMID: 26352407), [blood metabolite levels](http://www.ebi.ac.uk/gwas/search?query=Blood%20metabolite%20levels) (PMID: 24816252), [mean platelet volume](http://www.ebi.ac.uk/gwas/search?query=Mean%20platelet%20volume) (PMID: 24026423, PMID: 22139419), [platelet count](http://www.ebi.ac.uk/gwas/search?query=Platelet%20count) (PMID: 22139419) |
| *OR51I1* | rs77336780 | C/G (A304G) | Hemolysis in sickle cell anemia (PMID: 23406172), upper lip thickness |
| *KLHDC2* | rs200121865 | G/C (G149A) | None |
| *DUS2* | rs202069030 | G/C (R51S) | None |
| *KANK2* | rs7188 | T/G | Total cholesterol (PMID: 26780889), [HDL cholesterol](http://www.ebi.ac.uk/gwas/search?query=HDL%20cholesterol) (PMID: 23505323), body height, brain fractional anisotropy |
| *N4BP2* | rs2271395 | A/G (T1587A) | [Platelet thrombus formation](http://www.ebi.ac.uk/gwas/search?query=Platelet%20thrombus%20formation) (PMID: 22550155), rheumatoid arthritis |
| Related to MI | | | |
| *CWH43* | rs202103723 | C/A (P511Q) | [Response to hepatitis C treatment](http://www.ebi.ac.uk/gwas/search?query=Response%20to%20hepatitis%20C%20treatment) (PMID: 22095909) |
| *STXBP2* | rs188212047 | G/T (L212F) | None |
| *CCHCR1* | rs1265110 | G/A | [Multiple myeloma](http://www.ebi.ac.uk/gwas/search?query=Multiple%20myeloma) (PMID: 23955597), [hematology traits](http://www.ebi.ac.uk/gwas/search?query=Hematology%20traits) (PMID: 23263863), [chronic obstructive pulmonary disease–related biomarkers](http://www.ebi.ac.uk/gwas/search?query=Chronic%20obstructive%20pulmonary%20disease-related%20biomarkers) (PMID: 23144326), [Stevens-Johnson syndrome and toxic epidermal necrolysis](http://www.ebi.ac.uk/gwas/search?query=Stevens-Johnson%20syndrome%20and%20toxic%20epidermal%20necrolysis%20(SJS-TEN)) (PMID: 21912425), [nevirapine-induced rash](http://www.ebi.ac.uk/gwas/search?query=Nevirapine-induced%20rash) (PMID: 21810746), anti–cyclic citrullinated peptide–positive rheumatoid arthritis, ulcerative colitis, body height, age-related macular degeneration, asthma, body mass index, Crohn's disease, ratio of forced expiratory volume to forced vital capacity, flucloxacillin-induced liver injury, glycated hemoglobin levels, prostate cancer, psoriasis, lipids (CH2, CH3, CH2CO, CH=CH*CH2CH2) |
| *UBXN11* | rs138559558 | G/A (R289C) | [Alzheimer’s disease and age of onset](http://www.ebi.ac.uk/gwas/search?query=Alzheimer%20disease%20and%20age%20of%20onset) (PMID: 26830138), [obesity-related traits](http://www.ebi.ac.uk/gwas/search?query=Obesity-related%20traits) (PMID: 23251661) |
| 10p12.2 | rs11007350 | C/T | [Severe influenza A (H1N1) infection](http://www.ebi.ac.uk/gwas/search?query=Severe%20influenza%20A%20(H1N1)%20infection) (PMID: 26379185), [3-hydroxypropylmercapturic acid and 3-hydroxy-1-methylpropylmercapturic acid levels in smokers](http://www.ebi.ac.uk/gwas/search?query=3-hydroxypropylmercapturic%20acid%20levels%20in%20smokers) (PMID: 26053186), [diisocyanate-induced asthma](http://www.ebi.ac.uk/gwas/search?query=Diisocyanate-induced%20asthma) (PMID: 25918132), [acute lymphoblastic leukemia (PMID: 23996088)](http://www.ebi.ac.uk/gwas/search?query=Acute%20lymphoblastic%20leukemia%20(B-cell%20precursor)), cannabis dependence (PMID: 21668797), age-related macular degeneration, amyotrophic lateral sclerosis, bipolar disorder, diabetic nephropathy in type 1 diabetes, earlobes, forced expiratory volume, rheumatoid arthritis |
| 6p21.3 | rs9258102 | T/C | [Age-related macular degeneration](http://www.ebi.ac.uk/gwas/search?query=Age-related%20macular%20degeneration) (PMID: 22694956), [nevirapine-induced rash](http://www.ebi.ac.uk/gwas/search?query=Nevirapine-induced%20rash) (PMID: 21810746), [chronic lymphocytic leukemia](http://www.ebi.ac.uk/gwas/search?query=Chronic%20lymphocytic%20leukemia) (PMID: 21131588), [nasopharyngeal carcinoma](http://www.ebi.ac.uk/gwas/search?query=Nasopharyngeal%20carcinoma) (PMID: 19664746) |
| *ROR2* | rs200867550 | C/T (V874I) | [Bone mineral density](http://www.ebi.ac.uk/gwas/search?query=Bone%20mineral%20density%20(hip)) (PMID: 26911590), [bipolar disorder (inflammation and infection response interaction)](http://www.ebi.ac.uk/gwas/search?query=Bipolar%20disorder%20(inflammation%20and%20infection%20response%20interaction)) (PMID: 25781172), [type 2 diabetes](http://www.ebi.ac.uk/gwas/search?query=Type%202%20diabetes) (PMID: 25483131), [bipolar disorder and schizophrenia](http://www.ebi.ac.uk/gwas/search?query=Bipolar%20disorder%20and%20schizophrenia) (PMID: 22688191), alcohol dependence, narcolepsy |
| 5q14 | rs9293471 | A/G | [Retinal vascular caliber](http://www.ebi.ac.uk/gwas/search?query=Retinal%20vascular%20caliber) (PMID: 21060863), Alzheimer's disease |
| 6p21.3 | rs439121 | G/T | [Age-related macular degeneration](http://www.ebi.ac.uk/gwas/search?query=Age-related%20macular%20degeneration) (PMID: 22694956), [nevirapine-induced rash](http://www.ebi.ac.uk/gwas/search?query=Nevirapine-induced%20rash) (PMID: 21810746), [chronic lymphocytic leukemia](http://www.ebi.ac.uk/gwas/search?query=Chronic%20lymphocytic%20leukemia) (PMID: 21131588), [nasopharyngeal carcinoma](http://www.ebi.ac.uk/gwas/search?query=Nasopharyngeal%20carcinoma) ((PMID: 19664746) |

Data were obtained from GWAS Catalog (http://www.ebi.ac.uk/gwas) or GWAS Central (<http://www.gwascentral.org/browser>). Phenotypes with *P* values of <0.001 are shown. PMID, PubMed ID; chr., chromosome.
